# Big Data and AI‐Powered Modeling: A Pathway to Sustainable Precision Animal Nutrition

**DOI:** 10.1002/advs.202507564

**Published:** 2025-09-26

**Authors:** Shuai Zhang, Changhua Lai, Jinbiao Zhao, Junjun Wang

**Affiliations:** ^1^ State Key Laboratory of Animal Nutrition and Feeding Ministry of Agriculture and Rural Affairs Feed Industry Centre College of Animal Science and Technology China Agricultural University Beijing 100193 P. R. China; ^2^ Frontiers Science Center for Molecular Design Breeding (MOE) China Agricultural University Beijing 100193 China

**Keywords:** artificial intelligence, big data, machine learning, mathematical model, precision animal nutrition

## Abstract

The global livestock production system faces significant challenges for sustainable development, including feed resource shortage and environmental pressures. Precision animal nutrition is crucial in addressing these challenges, in which the mathematical model is an indispensable tool. The traditional mathematical models exhibit certain limitations, particularly in accommodating the emerging demands of precision nutrition and feeding for individuals. New technologies, especially big data and artificial intelligence (AI), have shown great potential to mitigate the above shortcomings. This review has summarized the current landscape and applications of big data and AI‐powered modeling in animal nutrition and feeding, covering techniques including intelligent data acquisition, in vitro kinetics and multi‐omics data mining, data augmentation, advanced and explainable machine learning algorithms, multi‐objective and heuristic algorithms, and life cycle assessment‐based sustainability evaluation with case studies in pigs and alternative feed ingredients. Furthermore, this review has introduced the next‐generation model techniques, including those based on large language models, multi‐agents, and embodied AI robots, depicted the potential translation of the advancements from animal nutrition to human health, and discussed the limitations of AI‐powered modeling techniques. These pioneering techniques will provide new tools and paradigms for research and practices in animal nutrition and further promote animal husbandry's sustainable development.

## Graphical abstract

This review summarizes the current landscape of big data and AI‐powered modeling in animal nutrition, covering techniques including intelligent data acquisition, data augmentation, explainable machine learning, heuristic algorithms, and life cycle assessment‐based sustainability evaluation. Furthermore, this review introduces the next‐generation model techniques, such as those based on large language models and multi‐agents, and depicts the potential advancements in translation from animal nutrition to human health.

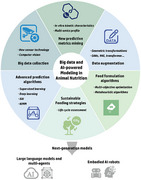

## 1 Introduction

Driven by global population growth and urbanization, the demand for animal‐source foods is expected to increase steadily. For instance, global meat consumption is anticipated to rise 12% by 2033 relative to the 2021–2023 base period, according to the Organization for Economic Co‐operation and Development (OECD) and the Food and Agriculture Organization (FAO).^[^ ^1^ ^]^ This escalating demand places significant pressure on the livestock production system, requiring it to operate more efficiently. Meanwhile, because the livestock sector contributes 14.5% of global greenhouse gas emissions, it is expected to develop toward a more environmentally friendly direction.^[^ ^2^ ^]^ Precision animal nutrition has appeared as a key strategy to improve the utilization efficiency of resources and reduce the environmental impact associated with livestock farming, in which mathematical models play a core role. However, conventional animal nutrition research and practices have long relied on established guidelines, empirical observations, and average responses across animal populations. Even though they are adequate to a certain extent, the traditional models cannot fully account for the complex interplay of factors influencing an animal's nutritional status and response to feeding, resulting in wasted resources, suboptimal performance, and potential health and environmental issues.

In recent years, the emergence of artificial intelligence (AI) has provided advanced analytical capabilities, especially in constructing and applying complex models, which usually require a big data foundation. Big data, characterized by its high volume, low veracity, high velocity, great variety, and high value,^[^ ^3^ ^]^ is usually collected through the Internet of Things (IoT) devices equipped with advanced sensors or high‐throughput sequencing techniques and handled with specialized computing infrastructure in a specialized environment.^[^ ^4^ ^]^ AI, including machine learning and deep learning, is a powerful tool for big data analysis and serves as the enabling power in driving the latest technological revolution and industrial transformation. Big data and AI have shown strong potential in analyzing complex biological systems, supporting predictive modeling efforts, and informing data‐driven decision‐making. As a result, their application in the livestock industry is considered a pivotal force in the digitalization, informatization, and intelligence process of the animal nutrition and feeding area and the entire livestock industry. Cases involving big data and AI‐powered modeling in the animal nutrition and feeding field have already been reported in prior literature.^[^ ^5^, ^6^ ^]^ The corresponding advancements may also be applied to clinical medicine, with precision animal nutrition as a blueprint for personalized human health interventions. However, there is still no systematic review focusing on the related subjects.

Therefore, this review aims to summarize the current landscape and applications of big data and AI‐powered modeling in animal nutrition and feeding, highlighting their advantages, key advancements, and future directions in this transformative field to further clarify the role of mathematical modeling in achieving sustainable precision animal nutrition. The topics covered in this review include intelligent data acquisition, in vitro kinetics and multi‐omics data mining, data augmentation, advanced and explainable machine learning algorithms, multi‐objective and heuristic algorithms, sustainability considerations, relevant case studies, next‐generation modeling approaches, the potential translation of these advancements from animal nutrition to human health, and the current limitations of the AI‐powered modeling technique. These pioneering techniques will provide new computational toolkits and paradigms for animal nutrition and feeding research and practices. They will further promote the sustainable development of animal husbandry in the future.

## 2 Challenges the Global Livestock Production System is Facing

As the primary provider of animal protein, the global livestock industry is facing significant challenges, with feed resource shortages and environmental pressures particularly prominent. Therefore, it is urgent to enhance production efficiency, optimize the utilization of limited resources, and reduce the environmental footprint of modern livestock production.

Intensive livestock production consumes about one‐third of the world's grains and two‐thirds of soybeans, corn, and barley.^[^ ^7^ ^]^ The competition between livestock and human consumption and biofuel production for feed resources threatens global food security. Feed shortages are driving up feed costs, making it more difficult for farmers and producers to maintain operations. Feeding grains and soybeans to animals for their meat and milk is less efficient regarding energy, protein, and land use than eating plants directly, which was also reported as an “opportunity food loss.”^[^ ^8^ ^]^ However, animal‐derived protein sources showed non‐substitutable superiority in their complete amino acid profile and greater bioavailability, establishing their indispensable role in achieving nutritional balance in human diets.^[^ ^9^, ^10^ ^]^ Hence, substantial space remains for improving feed efficiency in livestock production systems, which can directly reduce production costs and lower environmental pressure due to decreased nutrient excretion. Both genetic selection and nutritional management are direct strategies to enhance feed efficiency in livestock practices.^[^ ^11^, ^12^ ^]^ Exploring new feed resources and better utilization of non‐grain feed ingredients also serve as practical approaches to mitigate global feed resource scarcity.

Livestock production is a non‐negligible source of greenhouse gas emissions, especially for ruminant production, which includes methane from rumen fermentation and respiration, methane and nitrous oxide from manure management, and carbon dioxide from land use and its change.^[^ ^13^ ^]^ Moreover, the sustained growth in demand for animal‐source food has driven the expansion of livestock production scale, subsequently triggering increased demand for feed crops and pasture, which leads to deforestation and land use change, further exacerbating climate change.^[^ ^14^ ^]^ On the other hand, rising global temperatures and extreme weather events such as droughts, floods, and heatwaves are negatively impacting the production and quality of feed resources (crop and forage), water availability, animal health, productivity (reduced feed intake, lower milk production, increased mortality), reproduction, and biodiversity. They are pushing 1/3 of global food production outside safe climatic space.^[^ ^14^, ^15^ ^]^ Heat stress has emerged as a predominant factor receiving the most widespread attention and studies in recent years, with the potential to cause substantial economic losses, especially in the dairy field. In general, finding strategies to address the above challenges is the foundation for the sustainable development of livestock production, and it should also be a critical focus for future research in the animal husbandry field.

## 3 Precision Animal Nutrition is Crucial in Addressing the Above Challenges

The goal of precision animal nutrition (PAN) is to adjust nutrient supply according to the dynamic changes in animal nutritional requirements, preferably at the individual level, which involves the utilization of feeding techniques, such as nutritional modeling, alternative ingredients, and feed additives, that can provide a tailored nutrition profile to a group of animals promptly.^[^ ^16^ ^]^ Compared to traditional feeding methods, PAN considers the different nutrient requirements of individuals and focuses more on influential factors such as animal species, age, body weight, growth stage, health status, and environmental conditions. In some studies, PAN is also regarded as an important component of precision livestock farming (PLF), which applies the principles and technologies of process engineering to automatically and continuously monitor and control livestock farms.^[^ ^16^, ^17^, ^18^ ^]^ Unlike PLF, PAN emphasizes real‐time monitoring and intelligent management of feeds and the response of animals to nutrient supplies, which may also involve nutritional intervention strategies driven by data and models.^[^ ^17^ ^]^

Precision animal nutrition provides a promising solution for dealing with the challenges that global livestock production is facing, which include feed shortages, rising costs, environmental pressures, and climate change. For instance, previous studies have proven that the application of PAN in pig production reduced lysine intake, feeding costs, nitrogen and phosphorus excretion, and greenhouse gas emission by more than 25%, 8%, 40%, and 6%, respectively.^[^ ^19^, ^20^, ^21^ ^]^ First, PAN can maximize the nutritional values of available feed resources by improving its utilization efficiency, thereby reducing the total amount of feed required. Besides, PAN can make it possible to use cheaper alternative feed ingredients in feed formulations without compromising the nutrient requirements of the animals, directly reducing the total feed cost.^[^ ^22^ ^]^ Furthermore, PAN can assist in reducing feed waste and nutrient excretion by fully utilizing the ingested feed by the animals, thus lowering the risk of nutrient loss and environmental pollution and reducing greenhouse gas emissions from livestock farming, indirectly reducing the carbon footprint associated with feed production.^[^ ^17^, ^23^ ^]^ PAN can also alleviate the pressure of feed crop production on land and water resources by improving the production efficiency of animals and the utilization efficiency of agricultural by‐products.^[^ ^24^ ^]^ Lastly, PAN can help livestock better adapt to the challenges caused by climate change, such as heat stress, changes in feed supply, and increased disease incidence.^[^ ^25^, ^26^ ^]^ Precisely tailored animal diets can assist in coping with heat stress by adjusting energy density and nutrient balance,^[^ ^27^ ^]^ and optimal and balanced nutrition can aid in strengthening the immune system of animals, thereby enhancing their resilience and resistance to diseases that may become more prevalent due to climate change.^[^ ^28^ ^]^

## 4 The Role of Mathematical Modeling in Achieving Sustainable Precision Animal Nutrition

The mathematical model represents a biological process, system, or relationship using a mathematical equation or set of equations.^[^ ^29^ ^]^ It can be used to explain complex processes or to predict possible future trends.^[^ ^29^ ^]^ The mathematical model is an indispensable tool in precision animal nutrition, which can transform biological knowledge and data into a quantitative framework for understanding animal responses to nutrition, allowing for a more accurate prediction and optimization of nutrient bioavailability in feed, nutrient requirements of animals, and the complex interactions between animal and feed within livestock production systems.^[^ ^30^ ^]^ The development of mathematical models parallels the evolution of digital computing.^[^ ^30^ ^]^

### 4.1 Traditional Modeling Techniques in Animal Nutrition and Feeding

The application of mathematical models in animal nutrition and feeding dates back to the late 19^th^ century, with a history of evolving from simple equations describing animal growth and feeding standards (including feed evaluation systems and nutrient requirements) to more sophisticated systems that integrate various biological processes.^[^ ^30^ ^]^ The employment of mathematical models in traditional animal nutrition research can be roughly classified into three categories: animal growth models, feeding standard models, and animal response models. Animal growth models have expanded from simulating basic growth patterns of animals to quantifying digestive processes, metabolic pathways, lactation dynamics, egg production mechanisms, methane emission patterns, and digestion and fermentation kinetics. Feeding standard models have evolved beyond predicting feedstuff nutrient bioavailability to include comprehensive diet formulation optimization. Animal response models, marking a paradigm shift from descriptive to predictive modeling, now integrate performance forecasting (e.g., growth rates and feed efficiency), nutrient requirement estimation, feed intake prediction, body composition analysis, and holistic assessments of farming system impacts. These progressions from empirical, deterministic, and static models toward mechanistic, stochastic, and dynamic models with advanced computational frameworks can simulate changes over time and provide real‐time applications in precision animal nutrition. Next, we will introduce three commonly used mathematical models in traditional animal nutrition research.

#### 4.1.1 Modeling the Bioavailability of Nutrients in Animal Feed Ingredients

Determining available nutrients in feed ingredients relies on animal trials, which are usually costly and time‐consuming. For example, indirect calorimetry is regarded as the “golden standard” for net energy (NE) measurement,^[^ ^31^ ^]^ and the construction cost of related equipment typically reaches millions of RMB. Due to differences in climate conditions, geographical origins, cultivars, and processing techniques, significant variations exist in nutrient bioavailability among different sources of the same ingredient, necessitating the development of rapid prediction equations for practical applications. Given the limited accumulation of available nutrient data, traditional prediction models predominantly employ linear regression equations. Predictor selection prioritizes statistical significance and biological relevance,^[^ ^32^ ^]^ typically including proximate nutrient components, digestible constituents, and other available nutrients. Proximate nutrient components emerge as the most convenient predictors due to their marked variations across ingredients and sources and relatively simple analytical procedures. For instance, fiber components such as neutral detergent fiber (NDF), acid detergent fiber (ADF), and crude fiber are frequently negatively correlated predictors in predicting available energy values. At the same time, ether extract (EE) and starch often function as positive indicators. Notably, discrepancies may arise between statistically derived coefficients and biological principles during stepwise regression analysis. For example, a contradictory equation was developed for digestible energy (DE) prediction in rapeseed meal (DE = −1583 + 6.64 ash + 7.01 ADF − 33.17 NDF + 98.66 acid detergent lignin (ADL) + 1.07 gross energy (GE)),^[^ ^33^ ^]^ where positive coefficients for ADF and ADL conflict with the established negative correlation between fiber and available energy, illustrating methodological limitations of such approach. Advancements in chemical analysis have enabled more refined prediction methods. For instance, total dietary fiber (TDF) was used to predict DE and metabolizable energy (ME) in distillers dried grains with solubles (DDGS).^[^ ^34^ ^]^ At the same time, tannin content was incorporated in DE and ME prediction models in sorghum.^[^ ^35^ ^]^ Moreover, models with digestible constituents as predictors have further facilitated the development of in vitro simulated digestion technologies. In pigs, Dr. Jean Noblet pioneered the NE prediction models with digested nutrients as parameters, including digestible crude protein, digestible ether extract, starch (assumed fully digestible), and digestible residues, enabling mechanistic interpretation of energy metabolism.^[^ ^36^ ^]^

#### 4.1.2 Modeling the Nutrient Requirements of Animals

The empirical approach and the factorial approach primarily develop conventional nutrient requirement models. The empirical approach holistically estimates nutrient requirements through dose‐response feeding trials. While effective for group predictions under controlled conditions, this black‐box model lacks physiological interpretation and fails to elucidate individual requirements and their variation patterns, limiting its practical applicability. In these trials, linear, quadratic, or broken‐line models are established to quantify relationships between dietary nutrient levels and animal performance outcomes, generating early‐stage empirical models.^[^ ^37^ ^]^ The factorial approach partitions total nutrient requirements into different physiological components (e.g., maintenance, growth, production, lactation, pregnancy) for cumulative calculation. Despite its mechanistic advantages, implementation challenges for those mechanistic models include complex experimental procedures and the representativeness of sampled individuals.

Most early nutrient requirement models are deterministic static models focused on energy, protein/amino acids, and calcium/phosphorus, exemplified by CVB (Netherlands), INRA (France), and NRC (North America) systems. The feeding standards in China also predominantly rely on empirical models derived from the empirical approach. Since the 1980s, factorial‐derived mechanistic models have gained prominence by elucidating nutrient partitioning mechanisms and biological processes, especially in dairy and beef cattle. However, the establishment of mechanistic models for nutrient requirements in monogastric animals such as swine and poultry remains relatively underdeveloped, and comparative studies between empirical and factorial approaches remain scarce.

#### 4.1.3 Feed Formulation and Feeding Strategies Based on Mathematical Modeling

Feed formulation is a process of searching for the best feed ingredients combined with mathematical optimization to meet the nutrient requirements of animals and fulfill practical constraints such as minimizing total costs. Nowadays, the most widely used algorithms for feed formulation are linear programming (LP) and the simplex algorithm, which was first developed in 1947.^[^ ^38^ ^]^ The LP assumes the additivity of nutrient values of feed ingredients, with the lowest total feed cost as the only constraint, thus making it easy to compute. Nevertheless, LP may not always have a solution output, which is infeasible in multi‐objective optimization. Thus, other feed formulation algorithms have been developed, which include fuzzy linear programming (incorporating fuzzy constraints/objectives), nonlinear programming, and goal programming (introducing deviation variables and priority weights).^[^ ^39^, ^40^ ^]^ Among those methods, nonlinear programming exhibits advantages in considering maximum profit by incorporating product prices into the formulation process. On the other hand, goal programming converts constraints into prioritized targets through deviation minimization, enabling multi‐objective optimization, which allows for the simultaneous consideration of various factors, such as minimizing nutrient variation while meeting production goals.^[^ ^22^ ^]^ Despite advancements in computer‐aided feed formulation software and algorithms, traditional LP remains the predominant algorithm in practical applications.

### 4.2 Limitations and Pitfalls in Current Precision Animal Models

Despite the significant advancements in precision animal nutrition, it is necessary to further address the limitations of current precision animal models to enhance their effectiveness and robustness. Accurate modeling in precision animal nutrition requires comprehensive, high‐quality data on animal physiology, behavior, and production responses.^[^ ^17^ ^]^ However, restricted by the low efficiency and high cost of the conventional animal nutrition approaches, insufficient data volume and limited predictive metrics are significant pitfalls in the data collection process, resulting in static and simple model formats, with limited ability to learn from new data and adapt to changing conditions.^[^ ^41^ ^]^ In most cases, simple linear models have limited integration of real‐time data from sensors and other sources, making it difficult to make dynamic adjustments and falling short in using artificial intelligence and machine learning techniques to discover hidden patterns and improve prediction accuracy.^[^ ^42^ ^]^ With the evolution of livestock production, new data from various sources are constantly emerging. Although linear regression is not necessarily inferior to machine learning‐based algorithms in model development, considering the prediction accuracy, it is tricky for traditional models to incorporate the new information automatically. Thus, developing new precision animal models is urgent, especially for those powered by AI techniques.^[^ ^43^, ^44^ ^]^

## 5 Big Data and AI‐Powered Modeling in Animal Nutrition and Feeding

**Figure 1:**
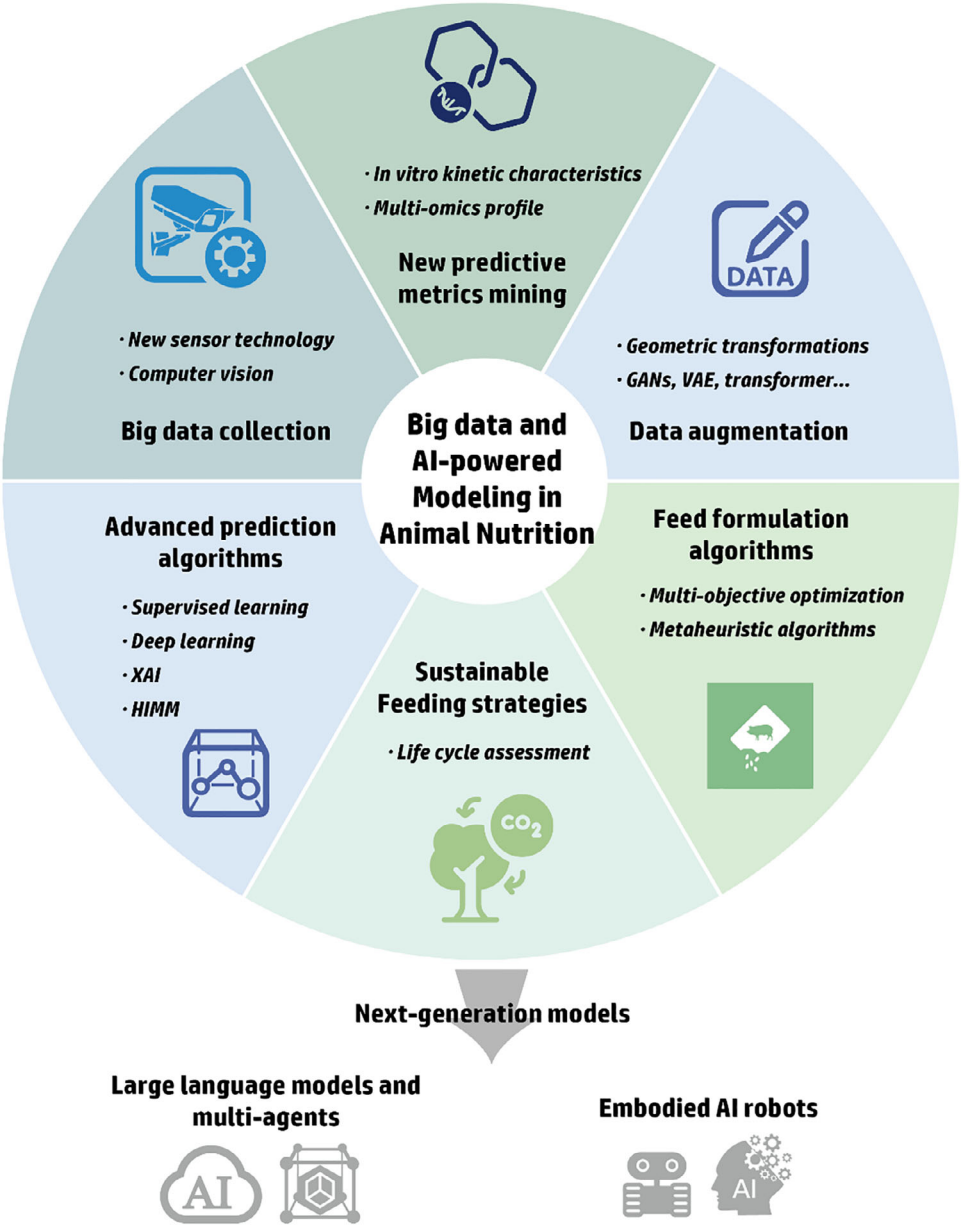
Schematic of the big data and artificial intelligence (AI)‐powered modeling in animal nutrition and feeding. GANs: generative adversarial networks; HIMM: hybrid intelligent mechanistic models; VAE: variational autoencoders; XAI: explainable artificial intelligence.

Powered by emerging technologies in AI, there is a significant trend of increased data‐driven models integrating with traditional knowledge‐driven models to enhance the accuracy and applicability of nutritional management strategies.^[^ ^18^ ^]^ In reality, AI models require big data to realize their full potential, making it vital to generate and scale up high‐throughput datasets by techniques such as an on‐site monitoring system. In turn, big data requires AI‐assisted tools for efficient and meaningful analysis, which relies on the development of powerful modeling algorithms, with the final goal to achieve more tailored and effective feeding regimes in sustainable livestock production. The schematic of the big data and AI‐powered modeling in animal nutrition and feeding is shown in **Figure** **1**.

### 5.1 Intelligent Sensors and Computer Vision‐Driven Big Data Collection

New sensor technology has laid a solid foundation for big data collection in animal nutrition and feeding areas. Various sensors, such as wearable, environmental, and image‐based, have revolutionized how data is collected from livestock production.^[^ ^45^, ^46^, ^47^ ^]^ Researchers have even created ingestible sensors that can measure physiological parameters in the animal body, including pH, gas, and biomarker sensors.^[^ ^48^, ^49^ ^]^ Traditional data collection methods rely heavily on infrequent manual measurements. However, new sensor technologies can continuously generate real‐time and high‐resolution data, providing more evidence for how animals respond to feed and nutrient supplies.

Standard wearable sensors include accelerometers, GPS trackers, temperature sensors, heart rate monitors, and respiratory rate monitors, which are usually embedded in ear tags, collars, jackets, or leg rings. Wearable sensors can be applied to gather data on activity levels, location, body temperature, and physiological responses of animals, offering insights into the animal's behavior, health, nutritional status, and even reproductive cycle.^[^ ^50^ ^]^ Environmental sensors can be applied within livestock housing to monitor key environmental factors such as temperature, humidity, and gas composition (e.g., ammonia levels) to ensure the maintenance of optimal environmental conditions for animals. In‐line sensors are usually integrated into feeding and watering systems, which measure parameters such as feed consumption, water intake, and even the electrical conductivity of milk in robotic milking systems.^[^ ^50^ ^]^ Bolus sensors are used by indigestion to measure parameters within the digestive tract, such as gastrointestinal pH and temperature, offering direct insights into the internal physiological state and the digestion characteristics of the animal.^[^ ^50^ ^]^ Moreover, radio‐frequency identification (RFID) tags are attached to livestock to associate the sensor data with specific animals to facilitate individual animal identification and tracking.^[^ ^51^ ^]^

In addition to measuring animal‐related parameters, intelligent sensor devices such as spectroscopy can rapidly determine nutrient values in feed or feed ingredients. Spectroscopic technology has fundamentally reduced dependence on wet‐chemical‐based experiments in the laboratory for nutrient analysis, significantly advancing rapid on‐site nutrient assessment of feed or feed ingredients. Near‐infrared (NIR) spectroscopy is currently the predominant technique in the feed industry. With NIR spectroscopy, chemical bonds in organic molecules (e.g., C─H, O─H, N─H) exhibit stretching and bending vibrations within specific spectral regions (wavelength range: 780–2500 nm),^[^ ^52^ ^]^ the mode of which has strong correlations with the content of organic components in feed ingredients, including proteins, lipids, and carbohydrates.^[^ ^53^ ^]^ Based on this principle, mathematical models can be established between spectral data and the referred wet chemistry analytical values, thus enabling rapid determination of nutrient properties in feed or feed ingredients.^[^ ^53^ ^]^ Moreover, mid‐infrared (MIR) spectroscopy (wavelength range: 2500–25000 nm) has also been implemented in some studies, which exhibits enhanced anti‐interference capability in resolving overlapping absorption signals, thus exhibiting superior resolution in complex mixtures.^[^ ^54^ ^]^ For instance, applying Fourier‐transform MIR accurately determined fibrous components in plant biomass and forage feed, including cellulose, hemicellulose, and lignin.^[^ ^55^, ^56^ ^]^ Although NIR spectroscopy has been widely used in feed analysis, its application remains constrained by the prerequisite for homogeneous sample grinding to minimize spectral interference. Furthermore, the limited spectral coverage of NIR spectroscopy cannot fully capture all feed characteristics. As a result, hyperspectral imaging (HSI) technology has recently been introduced into rapid feed evaluation.^[^ ^57^ ^]^ HSI integrates machine vision with spectral analysis and can simultaneously capture spatial and spectral information, thus generating 3D hypercube images (containing two spatial dimensions and one spectral dimension).^[^ ^57^ ^]^ This innovation presents novel possibilities for complex component analysis in feed ingredients.

Computer vision technologies are another powerful tool for large amounts of data collection in livestock production. Cameras can monitor various animal aspects without direct contact, and sophisticated image processing algorithms lay a solid foundation for data analysis.^[^ ^58^ ^]^ In animal nutrition studies, computer vision is usually used to monitor animals’ feeding and drinking behavior. Moreover, computer vision can also be applied for feed intake estimation by analyzing images of feeders or individual feeding stations before and after animal access, which is an excellent alternative to manual weighing.^[^ ^49^, ^58^ ^]^ To achieve greater accuracy, some studies used depth sensors with RGB cameras and convolutional neural networks (CNNs) in individual feed intake prediction.^[^ ^59^ ^]^ Computer vision can also estimate body weight in experimental conditions, especially for animals with relatively large body masses, such as cattle and pigs.^[^ ^60^, ^61^ ^]^ Although the large‐scale application of these technologies under practical production conditions still requires further investigation, the noninvasive estimation of animal body weight, feed intake, and body composition through computer vision offers novel pathways for establishing and practically implementing precision nutrition models. When cameras are installed in feeding or drinking areas, computer vision algorithms can be used to track the behaviors of animals, including feeding behavior (the frequency and duration of feeding events), rumination behavior in ruminant animals, drinking behavior, and behaviors within a herd or flock.^[^ ^62^, ^63^ ^]^ Computer vision can also be used to estimate the body condition score, fat reserves, and muscle mass of animals,^[^ ^64^ ^]^ which can provide valuable information for feed formulation optimization. The representative techniques and sensors for data collection in PAN‐related research are summarized in **Table** **1**.

**Table 1:** Summary of the representative techniques and sensors for data collection in precision animal nutrition (PAN)‐related research.

Data recorded for PAN^a)^	Technology	Radar chart of accuracy (A), economic efficiency (B), and operability (C)	Advantages	Limitations	Applied species
Body weight (for animal nutrient requirements and growth performance evaluation)	2D camera (2D image data)	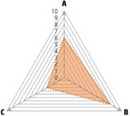	Non‐invasive High‐throughput Low cost	Rely on the lighting conditions Require efficient algorithms	Pig, cattle, sheep
	3D depth camera (3D cloud point data)	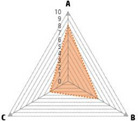	Non‐invasive High‐throughput	Rely on the lighting conditions Require extensive computational resources and efficient algorithms	Pig, cattle, sheep
	Automatic weighing	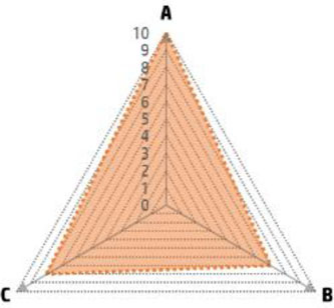	High accuracy (golden standard)	Require unique installation locations (e.g., walk‐through) High wear and tear on equipment	Pig, cattle, sheep, poultry
	Pressure exerted by the front legs	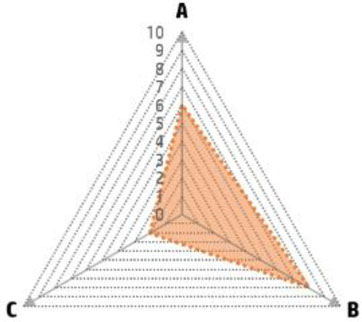	Easy operation	Unstable data acquisition Require efficient algorithms Relatively low accuracy	Pig, cattle, sheep
Body composition (for animal nutrient requirements and growth performance evaluation)	Doubly labeled water	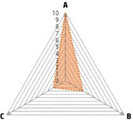	High accuracy (golden standard)	High cost Require specialized equipment for measurement	Pig, cattle, sheep, poultry
	Comparative slaughter	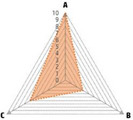	High accuracy	Non‐repetitive High cost Labor‐intensive	Pig, cattle, sheep, poultry
	Digestive and metabolic trial, indirect calorimetry trial, and carbon‐nitrogen balance technique	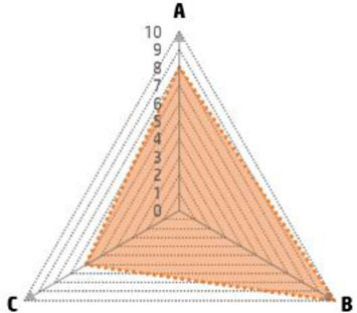	High accuracy Relatively low cost	Labor‐intensive	Pig, cattle, sheep, poultry
	Ultrasound	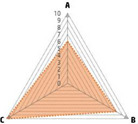	Easy operation Non‐invasive High‐throughput Relatively low cost	Low accuracy	Pig, cattle, sheep
	Medical imaging (Dual‐energy X‐ray absorptiometry, DXA/Computer tomography, CT/Magnetic resonance imaging, MRI)	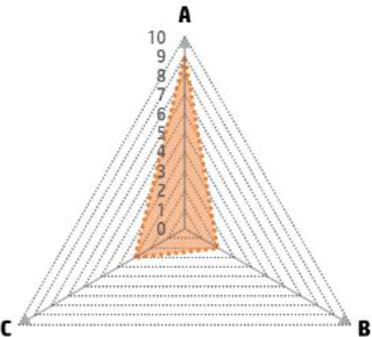	Non‐invasive High accuracy High‐throughput	High cost, especially for the specialized equipment Require professional operation Animals require anesthesia High radiation dose may raise safety concerns	Pig, cattle, sheep, poultry
	Bioelectrical impedance analysis (BIA)	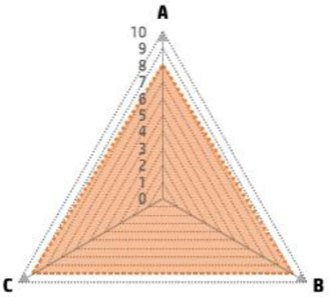	Easy operation Non‐invasive Portable equipment Relatively low cost High‐throughput	Require the development of specialized mathematical models, which may be affected by factors such as breed, body weight, and health status	Pig, cattle, sheep, poultry
Feed intake (for growth performance evaluation)	Electronic feeding system	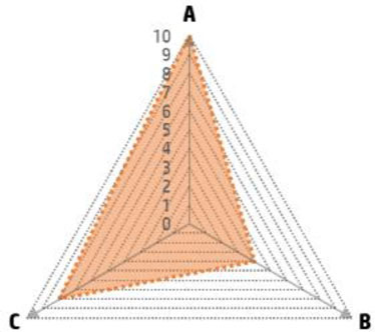	High accuracy	Relative high cost High wear and tear on equipment	Pig, cattle, sheep, poultry
	RGB camera (image data)	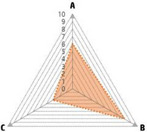	Non‐invasive High‐throughput	Rely on lighting condition Require efficient algorithms	Pig, cattle, sheep, poultry
Water consumption (for growth performance evaluation)	Water meter	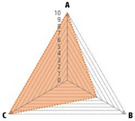	High accuracy	High wear and tear for equipment	Pig, cattle, sheep, poultry
	RGB camera (image data)	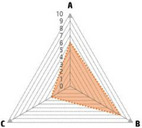	Non‐invasive High‐throughput	Rely on the lighting conditions Require efficient algorithms	Pig, cattle, sheep, poultry
Bioavailable nutrients in feed ingredients	Animal trial and wet chemical analysis	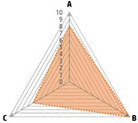	High accuracy (golden standard)	Labor‐intensive Require specialized equipment for measurement	/
	Near‐infrared (NIR) and mid‐infrared (MIR) spectroscopy	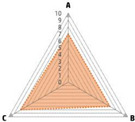	Non‐invasive High‐throughput Portable equipment	Require the development of specialized mathematical models	/
	Hyperspectral imaging (HSI)	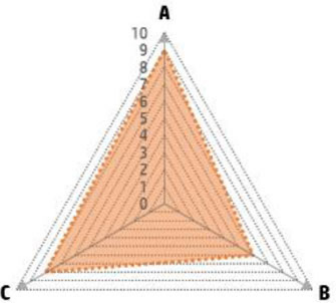	Non‐invasive High‐throughput Portable equipment More accurate than NIR and MIR	Require the development of specialized mathematical models	/
Activity (for animal nutrient requirements evaluation)	Global positioning system (GPS) sensors	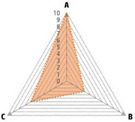	High accuracy High‐throughput	Relative high cost	Cattle, sheep
	Camera (image data)	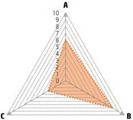	Non‐invasive High‐throughput	Rely on the lighting conditions Require efficient algorithms	Pig, cattle, sheep
	Accelerometer	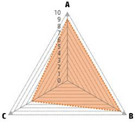	High accuracy High‐throughput	Require the development of specialized mathematical models	Pig, cattle, sheep
Physical status (for animal nutrient requirements evaluation)	Thermal infrared camera (temperature)	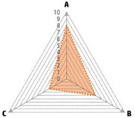	Non‐invasive High‐throughput	Rely on the lighting conditions Require efficient algorithms	Pig, cattle, sheep
	Bolus (internal sensors)	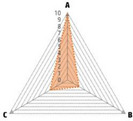	High accuracy High‐throughput	High cost Require specialized equipment for measurement	Cattle
Identification (for precision individual nutrition)	Radio‐frequency identification (RFID) ear tag	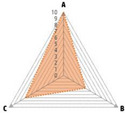	High accuracy	Relative high cost	Pig, cattle, sheep
	Ear tag with quick response (QR) code	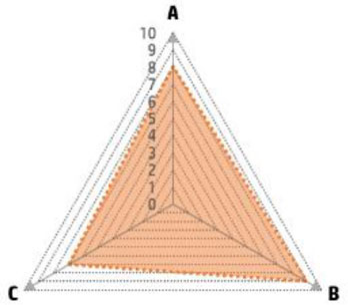	Relative low cost	Prone to wear and contamination	Pig, cattle, sheep

^a)^ Sources are mainly retrieved, screened, and summarized from Borges Oliveira et al. (2021), Gaillard et al. (2021), Odintsov Vaintrub et al.(2021), Tzanidakis et al. (2021), Zhang et al.(2021), Liu et al. (2023), Hlimi et al. (2024), and Wang et al. (2024).^[^ ^128^, ^129^, ^130^, ^131^, ^132^, ^133^, ^134^, ^135^ ^]^

However, applying intelligent sensor and computer vision technology in a broader scope is still challenging, even with significant advancements. First, installing and maintaining sensor networks requires a high cost, and the smaller‐scale producers may be unable to afford them. Moreover, various lighting conditions in different farms can produce bias when analyzing image results. Besides, processing large volumes of image data requires extensive computational resources and efficient algorithms, and data integration from various sensor sources into a unified platform is also a crucial challenge.^[^ ^65^ ^]^ In addition to advancements in more robust, cost‐effective, and user‐friendly sensors, deep learning models, edge computing, and standardized data protocols and platforms will provide potential solutions for the above challenges.^[^ ^65^ ^]^ It is promising that continuous and large‐volume data provided by intelligent sensors and computer vision will further facilitate AI‐driven precision animal nutrition models and strategies.

### 5.2 New Predictive Metrics Mining from In Vitro Kinetic Characteristics of Feed Ingredients and Multi‐Omics Profiling in Animals

In addition to traditional animal nutrition indicators (nutrient bioavailability indexes of feed ingredients and physiological parameters of animals such as body weight), new animal nutrition evaluation indicators are emerging, including in vitro digestive and fermentative characteristics of the feed ingredients and biomarkers of nutritional status and health in animals.

Current research on nutrient balance usually emphasizes the molecular structures and compositions of individual nutrient elements, especially carbohydrates, amino acids (AAs), and fatty acids, because they are analyzed through relatively stable methodologies. However, the full growth potential of animals often remains unrealized due to interactions between macronutrients, even when diets meet recommended nutritional standards.^[^ ^66^ ^]^ Previous studies have highlighted significant interaction effects between carbohydrates and protein and fat and protein in pigs and calves, indicating the importance of keeping nutrient combinations balanced in dietary formulations.^[^ ^67^, ^68^ ^]^ Achieving dynamic equilibrium between those nutrients requires synchronized glucose and AA release patterns. Especially for neonatal pigs, glucose derived from starch digestion is the primary energy source for vital functions, enabling enhanced AA absorption and utilization for protein synthesis rather than oxidation. This synchronization improves protein deposition and efficiency while conserving energy resources.^[^ ^69^ ^]^ Consequently, digestive and fermentative kinetics can be integrated into the modern feed evaluation system to optimize spatiotemporal nutrient synchronization. Thus, combining in vitro analyses of digestion and fermentation kinetics with in vivo nutrient evaluation of feed ingredients can offer a more precise assessment framework. For instance, precision models were developed to predict the in vivo organic matter digestibility and ME and NE values in ruminant diets, and the digestive and fermentation characteristics of feed ingredients from 400 in vitro experiments significantly improved the prediction accuracy.^[^ ^70^, ^71^ ^]^ These findings illustrate that in vitro kinetic characteristics of feed ingredients play a vital role in feed nutrient evaluation as a predictive metric.

Furthermore, as the cost of high‐throughput sequencing declines, an increased amount of multi‐omics data appears in the animal nutrition research area to assist in gaining deeper insights into the complex biological processes underlying nutrient utilization and animal performance. Genomics, transcriptomics, proteomics, and metabolomics are standard omics technologies that can provide biological information from different layers and offer a more holistic understanding of the animals when integrated.^[^ ^72^ ^]^ Genomic variation data, such as single‐nucleotide polymorphisms (SNPs) and other genetic variants, provide a foundation for identifying molecular markers associated with nutrition‐related traits, including growth rate, meat quality, feed efficiency, and disease resistance. Transcriptomic profiling enables the discovery of differentially expressed genes and pathways, offering mechanistic insights into nutritional responses at the gene regulation level. Proteomic data capture functional protein changes resulting from dietary interventions, helping elucidate post‐transcriptional regulation. Metabolomic profiling reflects the biochemical state of an organism under specific physiological conditions and can be leveraged to identify metabolic biomarkers that inform individual nutrient requirements and metabolic health. Besides, changes in the microbiome and the diet‐microbiota‐host axis are also commonly analyzed in current animal nutrition and feeding research.^[^ ^73^ ^]^ Multi‐omics data from different modalities can be integrated and analyzed, allowing for a more comprehensive understanding of the complex interactions between different modalities in response to nutrition.^[^ ^74^ ^]^ Novel biomarkers can be identified, and more accurate models can be developed by mining predictive metrics from these integrated multi‐omics profiles. However, each modality offers distinct and sometimes non‐overlapping information. Certain biological signals or regulatory features detectable in one modality (e.g., transcriptomics) may not be captured in another (e.g., proteomics). Therefore, integrative analysis requires careful consideration of these modality‐specific limitations and complementarities. Additionally, multi‐omics datasets are often large, heterogeneous, and generated from different platforms. Integrative analysis always requires sophisticated bioinformatics tools and statistical methods. Strategies to reduce the dimensionality of these high‐dimensional datasets and translate the complex mechanisms and findings into practical solutions should also be planned when applying those new metrics.

### 5.3 Data Augmentation Based on Machine Learning Algorithms

Machine learning models, especially deep learning models, often require large amounts of data to train effectively. However, the conventional data collection approach relies on animal trials, which are usually expensive and time‐consuming. The limited number of animals available and the complex experimental designs directly lead to data scarcity, resulting in overfitting when machine learning algorithms are applied, with a performance that models perform well on the training data but fail to generalize to new, unseen data.^[^ ^75^ ^]^ Data augmentation techniques potentially solve this problem by artificially creating variations on the existing data or synthesizing new data. With their help, the size of training datasets will enlarge, and the machine learning models will become more robust with an improved ability to generalize to real‐world scenarios.^[^ ^76^ ^]^ Depending on the data types, various machine learning algorithms can be employed for data augmentation in animal nutrition research.

Geometric transformations are commonly used to augment image data obtained from machine vision systems. These methods include rotation, translation, scaling, and flipping of images.^[^ ^76^ ^]^ Statistical methods such as bootstrapping can be used for omics data and sensor data, which involves resampling the original data with replacement to create multiple slightly different dataset versions.^[^ ^77^ ^]^ Random noise injection to existing data points can also increase the variability and improve the model's resilience to noisy real‐world data.^[^ ^77^ ^]^ For time‐series data from sensors, techniques such as time series flipping, time series combination, magnitude warping, or time series interpolation can be used to generate augmented data.^[^ ^77^ ^]^ In addition, more advanced techniques can be used to generate synthetic data by learning underlying data distributions and patterns from real‐world datasets, such as generative adversarial networks (GANs), Bayesian models, variational autoencoders (VAE), diffusion models, and transformer‐based architectures.^[^ ^78^, ^79^, ^80^, ^81^ ^]^

Due to the introduction of unrealistic data variations, data augmentation techniques may also negatively influence model performance, such as overfitting. As a result, it is important to set up screen criteria and double‐check the synthesized datasets when data augmentation techniques are applied in animal nutrition research. Moreover, other potential techniques may handle the data sparsity problem, including transfer learning and collaborative data‐sharing strategies such as federated learning, which can enhance machine learning models’ generalization capacity.^[^ ^82^, ^83^ ^]^

### 5.4 Advanced and Explainable Machine Learning Algorithms in Feed Nutrients and Animal Nutrient Requirements Prediction

The machine learning algorithms can be applied based on sufficient data collection through sensors, computer vision, in vitro kinetics, and omics. Compared to the traditional simple and multiple linear regression models, machine learning models have the potential to automatically uncover latent patterns in multidimensional and large‐scale datasets, and may achieve more accurate prediction results. These models may also exhibit superior adaptability by incorporating different variables into a unified computational structure, such as animal genetic backgrounds, physiological states, disease factors, and environmental conditions, which can better elucidate the intricate interactions between feed supplies and animal responses.

From a learning paradigm perspective, machine learning can be categorized into supervised, unsupervised, semi‐supervised, and reinforcement learning.^[^ ^84^ ^]^ Given that predicting feed nutrient bioavailability or animal nutrient requirements inherently falls under regression problems, supervised regression algorithms such as decision tree regression, random forest regression, gradient boosting machines, neural networks, and Bayesian regression are typically used. Notably, reinforcement learning could be strategically implemented during model deployment phases to enable dynamic dietary formulation adjustments through continuous environmental feedback.^[^ ^84^ ^]^ Data characteristics and research objectives are guidelines for algorithms in practice, with potential synergistic combinations of these approaches. For instance, ensemble methods integrating decision trees with gradient boosting often enhance predictive robustness. At the same time, hybrid architectures combining neural networks with Bayesian frameworks may optimize both predictive power and uncertainty estimation.^[^ ^85^, ^86^ ^]^ In addition, deep learning models, including convolutional neural networks (CNNs) and recurrent neural networks (RNNs), have succeeded in analyzing image and time‐series data, respectively.^[^ ^84^ ^]^ However, those models have been primarily used in animal behavior identification, such as using CNN and long short‐term memory (LSTM) models to extract features of drinking behavior.^[^ ^87^ ^]^ Integrating these data with nutritional profiles to achieve precision feeding remains a subject warranting further investigation. The representative advanced machine learning algorithms used in mathematical model development in PAN research are summarized in **Table** **2**.

**Table 2:** Summary of the representative machine learning algorithms used in mathematical model development in precision animal nutrition research.

No.	Objectives^a)^	Machine learning algorithms involved ^b)^	Data sources	The best algorithms or prediction metrics ^c)^	Refs.
1	Predicting the growth performance and feed efficiency in mink	DT, GTB, LR, LSVM, SVR, RFR, and XGBoost	Collected from an animal trial (traditional methods)	XGBoost provided the most accurate prediction for ADG (R^2^ = 0.71, RMSE = 0.10 kg), FCR (R^2^ = 0.74, RMSE = 0.14), and RFI (R^2^ = 0.76, RMSE = 0.10 kg).	Shirzadifar et al. (2025)^[^ ^136^ ^]^
2	Estimating the metabolizable protein supply from feed in lactating dairy cows	RFR and SVR	Merging two published and peer‐reviewed databases	RFR model performed better in predicting rumen‐undegradable protein (R^2^= 0.60, RMSE = 0.326 kg d^−1^, CCC = 0.71); SVR model performed better in predicting duodenal microbial nitrogen (R^2^ = 0.76, RMSE = 42.4 g d^−1^, CCC = 0.86).	Lee et al. (2025)^[^ ^137^ ^]^
3	Predicting the dry matter intake in growing Black Bengal goats	ANN	Collected from an animal trial (traditional methods)	R^2^ = 0.9693; MSE = 0.0013 kg d^−1^	Singh et al. (2025)^[^ ^138^ ^]^
4	Predicting the net energy requirements in growing‐finishing pigs	ANN, KNN, MLR, and RFR	Collected from several animal trials (traditional methods)	ANN models demonstrated the smallest RMSE and the largest R^2^.	Yang et al. (2024);^[^ ^108^ ^]^ Wang et al. (2023)^[^ ^6^ ^]^
5	Predicting the feed efficiency in dairy sheep	ANN, multivariate regression, RFR, SVR, and XGBoost	Milk metabolites	The orthogonal partial least squares algorithm outperformed other algorithms for FCR prediction.	Marina et al. (2024)^[^ ^139^ ^]^
6	Predicting the daily nutrient requirements in gestating sows	GTB, KNN, LASSO regression, LR, MLP, PR, RFR, RR, and SVR	Electronic feeders and drinker stations, connected weight scales, accelerometers, and cameras	The GTB model showed the lowest RMSE (0.91 MJ d^−1^ for energy and 0.08 g d^−1^ for lysine).	Durand et al. (2023)^[^ ^5^ ^]^
7	Predicting the body composition in growing‐finishing pigs	MLR, RFR, and SVR	Ultrasound‐based back‐fat depth measurement	SVR outperformed MLR and RF models in predicting pigs’ fat mass and fat‐free mass.	Basak et al. (2023)^[^ ^140^ ^]^
8	Predicting the fiber digestibility in Holstein dairy cows	ANN	Merged data from 11 previous studies	R^2^ = 0.90, RMSE = 3.26%	Cavallini (2023)^[^ ^141^ ^]^
9	Predicting the drinking water requirements in growing‐finishing pigs	ENET, MLP, MLR, RFR, and SVR	Collected from two animal trials (traditional methods)	The RFR algorithm performed the best, and the MLR methods performed the worst.	Basak et al. (2023)^[^ ^142^ ^]^
10	Predicting the dry matter intake in beef cattle	RFR, RMANOVA for full or core algorithms, RMRF	In‐pen‐weighing positions and feed intake nodes	The RMRF algorithm provided the slightest prediction error (RMSE = 0.75 kg).	Blake et al. (2022)^[^ ^143^ ^]^
11	Predicting the precision feed intake in dairy cows	BP, KNN, logistic regression, multilayer BP, and SVR	Smart collar device	The BP model using the polynomial decay learning rate performed the best model with R^2^ = 0.94.	Shen et al. (2022)^[^ ^144^ ^]^
12	Predicting the precision feed intake in dairy cows	CNN, MLP‐CNN, and TL‐CNN	Red‐Green‐Blue‐Depth (RGBD) camera	TL models performed best (MAE = 0.12–0.13 kg, RMSE = 0.17–0.18 kg).	Saar et al. (2022)^[^ ^145^ ^]^
13	Predicting the pellet quality of pelleted feeds	ABR, DT, GTB, KNN, LASSO regression, LR, LSVM, MLP, RFR, RR, SR, and SVR	Collected from a modern plant	The SVR algorithm was the best model for predicting the pellet durability index (MAE = 3.280, MSE = 16.192, CCC = 0.636).	You et al. (2022)^[^ ^146^ ^]^
14	Predicting the precision feed intake in dairy cows	ABR, DT, ETR, GTB, KNN, LR, LSVM, MLP, NuSVR, RFR, RR, and SVR	Jaw movement using a triaxial accelerometer	The ETR algorithm was the best model for predicting feed intake (R^2^ = 0.97, RMSE = 0.36 kg, NME = 0.05).	Ding et al. (2022)^[^ ^147^ ^]^
15	Predicting the dry matter intake in Canadian Holstein dairy cattle	ANN	Milk mid‐infrared (MIR) spectroscopy	ANN using Bayesian regularization and scaled conjugate gradient training algorithms yielded better weekly average dry matter intake predictions.	Shadpour et al. (2022)^[^ ^148^ ^]^
16	Evaluating the metabolizable energy requirements in dairy cows	Linear extrapolation, orthogonal polynomials of third order, regression models	Proximal hyperspectral sensing coupled with a canopy pasture probe system	The estimated daily ME requirement varied greatly from the actual ME supplied.	Duranovich et al. (2021)^[^ ^149^ ^]^
17	Predicting the feed intake in finishing beef steers	MLR, RFR, and SVR	Electronic feeding equipment and recorded feeding behavior	Although machine learning models produced lower errors associated with individual intakes, the overall prediction precision was too low for practical use.	Davison et al. (2021)^[^ ^150^ ^]^
18	Predicting the feed efficiency in growing pigs	GTB and RFR	Merged whole blood transcriptomic datasets	GTB models were more accurate in predicting FCR (R^2^ = 0.80, RMSE = 0.08).	Messad et al. (2021)^[^ ^151^ ^]^
19	Predicting the precision feed intake in dairy cows	ResNet CNN	Red–Green–Blue‐Depth (RGBD) camera	MAE = 0.127 kg, MSE = 0.034 kg	Bezen et al. (2020)^[^ ^152^ ^]^
20	Predicting the average daily gain in pigs	ABR, DNN, DT, and LR	Synthetic dataset based on the theoretical growth model and measured data	DNN can provide a higher predictive accuracy.	Lee et al. (2019)^[^ ^153^ ^]^
21	Predicting the growth performance in pigs	DT, logistic regression, RFR, and SVR	Collected from 55 pig farms	DT and logistic regression showed the highest accuracy in ADG and feed intake prediction, respectively.	Lee et al. (2019)^[^ ^154^ ^]^
22	Predicting growth and carcass traits in pigs	Bayesian LASSO, GTB, RFR, and semi‐parametric kernel models	Collected from slaughter trial and microbiome data	The semi‐parametric kernel model (Reproducing Kernel Hilbert space) had the highest predictive power (0.32).	Maltecca et al. (2019)^[^ ^155^ ^]^
23	Predicting the dry matter intake in lactating dairy cows	ANN and PLS	Milk mid‐infrared (MIR) spectroscopy selected through Bayesian network	Using the Bayesian network combined with ANN yielded the highest accuracy and precision.	Dórea et al. (2018)^[^ ^156^ ^]^

^a)^ Sources are partially retrieved, screened, and summarized from Liu et al. (2023).^[^ ^133^ ^]^

^b)^ ABR: adaptive boosting regression; ANN: artificial neural network; BP: back propagation neural network; CNN: convolutional neural network; DNN: deep neural network; DT: decision tree; ENET: elastic net; ETR: extra trees regression; GTB: gradient tree boosting regression; KNN: K‐nearest neighbors; LASSO: least absolute shrinkage and selection operator; LR: linear regression; LSVM: linear support vector machine; MLP: multilayer perceptron; MLR: multiple linear regression; NuSVR: Nu support vector regression; PLS: partial least squares; PR: polynomial regression; ResNet: residual network; RFR: random forest regression; RMANOVA: repeated measures analysis of variance; RMRF: repeated measures random forest; RR: Ridge regression; SR: stacking regression; SVR: support vector machine for regression; TL: transfer learning; XGBoost: extreme gradient boosting.

^c)^ ADG: average daily gain; CCC: concordance correlation coefficient; FCR: feed conversion ratio; MAE: mean absolute errors; ME: metabolizable energy; MSE: mean square error; NME: normalized mean error; RFI: residual feed intake; RMSE: root mean square error.

Although machine learning models may exhibit excellent performance in prediction, they generally lack explanatory capability regarding the core relationships within the data, as their internal operational mechanisms remain opaque to users, often termed “black box models.”^[^ ^41^ ^]^ Model interpretability is crucial for understanding predictive outcomes. The lack of interpretability can reduce users’ trust and make debugging difficult when models exhibit errors or abnormal behavior.^[^ ^88^ ^]^ Explainable AI (XAI) techniques have been developed to solve the above problems. XAI aims to make the predictions of AI models more transparent and understandable to humans.^[^ ^89^ ^]^ Depending on whether it relies on specific model structures, it can be categorized into model‐agnostic and model‐specific approaches. The model‐agnostic method explains model behavior by analyzing input–output relationships without relying on specific model structures. This method can offer high flexibility but typically demands more extensive computational resources. In contrast, the model‐specific method generates explanations based on the internal structures of specific models, which can enhance efficiency but have limited flexibility. Furthermore, XAI can be divided into global and local explanations based on their scope. Global explanations elucidate the overall behavior of a model, helping users understand the general operational mechanisms of the model. Local explanations focus on individual predictions or specific prediction groups, helping users comprehend model decision‐making processes under specific circumstances. Currently, the most widely used model‐agnostic methods include partial dependence plots (PDP), individual conditional expectation (ICE), accumulated local effects (ALE), feature interaction, permuted feature importance, global surrogate models, local surrogate models (e.g., Local Interpretable Model‐agnostic Explanations, LIME), and Shapley values (SHAP).^[^ ^90^ ^]^

In addition to XAI methods, hybrid intelligent mechanistic models (HIMM) are a novel approach to enhance model transparency and interpretability, combining classical and AI‐driven models. HIMM consists of two parts: a data‐driven part that learns patterns and relationships from data (handling high‐dimensional, nonlinear, and complex interactions) and a mechanistic part based on knowledge (providing physical consistency, interpretability, and generalization to unknown scenarios).^[^ ^65^ ^]^ Some similar models or algorithms exist, such as physics‐informed neural networks (PINNs), gray‐box models, and knowledge‐guided machine learning.^[^ ^91^ ^]^

The importance of interpretability continues to grow as machine learning and AI models rapidly advance. Future research directions on model interpretability may include developing more efficient model‐agnostic methods, combining multiple XAI approaches, and improving the user‐friendliness of XAI tools. Additionally, open‐source code and documentation, and interactive human‐AI collaborative systems development are all potential future research directions to enhance transparency, verifiability, reproducibility, and practical utility of machine learning models.^[^ ^92^ ^]^

### 5.5 Feed Formulation Employing Multi‐Objective and Metaheuristic Algorithms

The goal of feed formulation is to meet specific nutritional requirements and production goals of animals. It is a complex task traditionally relying on linear programming techniques based on established feed nutrient tables and animal nutrient requirement standards. Even though LP is an effective calculation tool to meet nutrient requirements at a minimum cost, the economic objective may not be the only target the livestock industry is always pursuing.^[^ ^22^ ^]^ More objectives may be considered when formulating diets based on the livestock production practice, such as ensuring animal health and welfare, reducing environmental impact, and the availability and quality of feed ingredients. To address this problem, other approaches, such as multi‐objective optimization and metaheuristic algorithms, are introduced in animal feed formulation tasks.

Multi‐objective algorithms are designed to handle situations with several objectives that must be optimized simultaneously, with a final solution to find possible trade‐offs among different objectives.^[^ ^93^ ^]^ These algorithms are usually used when feed cost and environmental footprints must simultaneously be minimized in feed formulation. Multi‐objective optimization can be achieved by generating a Pareto efficiency frontier or trade‐off curve or directly setting the weight for various objectives.^[^ ^93^, ^94^, ^95^ ^]^ Metaheuristic algorithms are proposed in contrast to optimization algorithms to find a solution that is good enough for the given problem, especially when finding an exact optimal solution is difficult or expensive in computation. These algorithms are instrumental when solving complex feed formulation problems, such as nonlinear relationships and ingredient availability constraints. Most metaheuristic algorithms are nature‐inspired, and these include popular algorithms such as the genetic algorithm (GA), simulated annealing (SA), and particle swarm optimization (PSO).^[^ ^96^ ^]^ GA evaluates the individual based on a fitness function and uses an iterative approach to update a population, performing operations like crossover and mutation in genetics.^[^ ^96^ ^]^ SA is inspired by physical annealing, allowing an occasional uphill movement to avoid the problem of being stuck at local minima.^[^ ^96^ ^]^ PSO is an efficient algorithm for solving complicated optimization problems inspired by flocking behavior and the movement of birds.^[^ ^96^ ^]^ These algorithms have been applied to formulate poultry, cattle, and pig feeds.^[^ ^97^, ^98^, ^99^ ^]^ Furthermore, driven by the progress in AI technologies, machine learning algorithms such as artificial neural networks (ANNs) can also be potential decision‐making tools in precision feed formulation. The representative algorithms used in animal feed formulations are summarized in **Table** **3**.

**Table 3:** Summary of the representative algorithms and models used in animal feed formulations.

No.	Feed formulation algorithms/models^a)^	Animal species applied	Formulation objectives	Refs.
1	Combination spreadsheet	Unspecified	Minimize feed cost	Black and Hlubik (1980)^[^ ^157^ ^]^; VandeHaar and Black (1991);^[^ ^158^ ^]^ Thomson and Nolan (2001)^[^ ^159^ ^]^
2	Parametric linear programming	Beef cattle, dairy cow, broiler chicken, turkey	Minimize feed cost	Glen (1980);^[^ ^160^ ^]^ Crabtree (1982);^[^ ^161^ ^]^ Assis and France (1983);^[^ ^162^ ^]^ Glen (1986);^[^ ^163^ ^]^ Talpaz et al.(1986);^[^ ^164^ ^]^ Parmar et al. (1992)^[^ ^165^ ^]^; Al‐Deseit (2009)^[^ ^166^ ^]^
3	Iterative linear programming with multigoal	Dairy cow, broiler chicken, pig	Minimize feed cost	Rehman and Romero (1984);^[^ ^167^ ^]^ Lara and Romero (1992);^[^ ^168^ ^]^ Lara and Romero (1994);^[^ ^169^ ^]^ Munford (1996);^[^ ^169^ ^]^ Zhang and Roush (2000)^[^ ^170^ ^]^
4	Single‐chance constrained programming	Dairy cow	Minimize feed cost	St. Pierre and Harvey (1986)^[^ ^171^ ^]^
5	Joint‐chance constrained programming	Dairy cow	Minimize feed cost	St. Pierre and Harvey (1986)^[^ ^172^ ^]^
6	Mixed‐integer linear programming	Laying hen, dairy cow, broiler chicken	Minimize feed cost	Amir et al. (1978);^[^ ^173^ ^]^ Kleyn and Gous (1988);^[^ ^174^ ^]^ May et al. (1999);^[^ ^175^ ^]^ Chagwiza et al. (2016)^[^ ^176^ ^]^
7	Linear programming with the Markov decision process	Dairy cow	Minimize feed cost	Yates and Rehman (1998)^[^ ^177^ ^]^
8	Iterative quadratic programming	Unspecified	Minimize feed cost	Chen (1973)^[^ ^178^ ^]^
9	Nonlinear programming with other algorithms	Broiler chicken, sheep, dairy cow	Minimize feed cost	Guevara (2004);^[^ ^179^ ^]^ Saxena (2006);^[^ ^180^ ^]^ Li et al. (2022)^[^ ^181^ ^]^
10	Multi‐objective optimizations	Dairy cow, pig, broiler chicken, fish	Minimize cost and maximize the amounts of stored feeds, satisfying the economic, nutritional, and environmental requirements at the same time, e.g., reducing phosphorus concentration	Lara (1993);^[^ ^182^ ^]^ Mitani and Nakayama (1997);^[^ ^183^ ^]^; Castrodeza et al.(2005);^[^ ^184^ ^]^ Pomar et al.(2007);^[^ ^185^ ^]^ Tallentire et al.(2017);^[^ ^186^ ^]^ Mackenzie et al. (2017);^[^ ^187^ ^]^ Meda et al. (2021);^[^ ^95^ ^]^ Wilfart et al. (2022);^[^ ^188^ ^]^ de Quelen et al. (2024)^[^ ^189^ ^]^
11	Fuzzy programming	Unspecified	Minimize feed cost	Cadenas et al (2004)^[^ ^190^ ^]^
12	Multi‐objective stochastic programming	Unspecified	Minimize feed cost	Peña et al. (2009)^[^ ^191^ ^]^
13	Genetic algorithm/ Evolutionary algorithm	Shrimp, dairy cow, fish	Minimize feed cost	Abd Rahman et al. (2017);^[^ ^192^ ^]^ Uyeh et al. (2019);^[^ ^193^ ^]^ Soong et al. (2022)^[^ ^194^ ^]^
14	Particle swarm optimization algorithm	Unspecified	Minimize feed cost	Sahman et al. (2018)^[^ ^195^ ^]^
15	Multi‐objective Bayesian optimization algorithm	Pig	Minimize feed cost	Uribe‐Guerra et al. (2024)^[^ ^196^ ^]^

^a)^ Sources are mainly retrieved, screened, and summarized from Saxena and Chandra (2012), Saxena and Khanna (2014), and Akintan et al. (2024).^[^ ^22^, ^39^, ^40^ ^]^

### 5.6 Feeding Strategies Guided by Life Cycle Assessment

Evaluating the environmental impacts of different feeding strategies, such as feed ingredient choices and formulation, is crucial to achieving sustainable livestock production. Life Cycle Assessment (LCA) provides a comprehensive framework to assess the environmental footprints of a product or a system during its whole life cycle, also known as “from the cradle to the grave”. Applying LCA principles to animal feeding strategies allows a thorough evaluation of the environmental burdens associated with feed ingredient production, transportation, feed processing, animal production, and waste management. The standardized procedure for LCA includes identifying objectives and scope, the functional unit (FU), and system boundaries, building the life cycle inventory (LCI) database, conducting LCA to relate LCI data with potential environmental loads, and interpreting the results. The typical indexes of environmental effects LCA studies concern include carbon footprint, nitrogen footprint, global warming potential (GWP), land use, nonrenewable resource use (NRRU), acidification potential (AP), and eutrophication potential (EP).^[^ ^100^ ^]^

Several sustainable feeding strategies can be guided by LCA. Using low‐protein diets, high‐fiber ingredients, various additives such as feed‐use amino acids, substituting part of the soybean meal with other oilseed meals, or employing different feed formulation index systems, such as the net energy system, are all nutritional regulation measures that can reduce the environmental pressure of animal husbandry.^[^ ^100^, ^101^, ^102^, ^103^ ^]^ Combining LCA analysis with multi‐objective optimization algorithms can also reduce environmental emissions in livestock production. For example, in Canadian pig production, multi‐objective programming has been introduced into the feed formulation algorithm to reduce the environmental impacts of GWP, NRRU, AP, and EP.^[^ ^104^ ^]^ However, the relevant study did not consider the balance between economic and environmental costs, resulting in 12–30% greater economic cost of the optimized feed formulation than that of the traditional formulation.^[^ ^104^ ^]^ To address this issue, weighting coefficients were introduced to further optimize the feed formulations for European pigs, broilers, and beef cattle. The new algorithm for optimizing feed formulations has balanced economic and environmental costs well.^[^ ^93^ ^]^ The representative feeding strategies in different countries or regions that mitigate the environmental impacts of animal production are summarized in **Table** **4**. Because there are numerous relevant studies, this table summarizes only representative studies on pigs and chickens from recent years.

**Table 4:** Summary of the representative feeding strategies in different countries or regions that mitigate the environmental impacts of pig and chicken production evaluated by life cycle assessment.

Countries or regions	Animal species^a)^	Functional unit (FU)	Feeding or management strategies	Environmental impacts (CO_2_‐eq FU^−1^ unless otherwise specified)	Refs.
Argentina	Pig	1 t of CW	Maize, soybean, sorghum, sunflower, and wheat	9400 kg	Arrieta et al. (2022).^[^ ^197^ ^]^
	Pig and chicken	1 t of LW	Standard pig and chicken production	2.03–2.22 t for chicken and 5.14–6.45 t for pig	Arrieta and Gonzalez (2019).^198^ ^]^
Brazil	Pig	1 kg of BWG over the nursery stage	Low protein diets and synthetic amino acids supplementation	1.76–2.45 kg	Monteiro et al. (2019)^[^ ^199^ ^]^
	Chicken	1 kg of LW	Standard broiler chicken production	2.7 kg	Lima et al. (2019)^[^ ^200^ ^]^
	Pig	1 t of BWG at the farm gate	Precision feeding programs	1840 kg	Andretta et al. (2018)^[^ ^19^ ^]^
	Pig	1 kg of feed	Standard feed production	0.419–0.475 kg	Ali et al. (2018)^[^ ^201^ ^]^
Canada	/	/	30% wheat millrun	GPW decreased by 25%	Kpogo et al. (2021).^[^ ^202^ ^]^
China	Pig	1 kg of LW	Rapeseed meal, peanut meal, cottonseed meal, and extruded soybean	2.37–2.55 kg	Hu et al. (2023)^[^ ^100^ ^]^
	Pig	1 kg of LW	DDGS and oilseed meal	both 1.4 kg	Long et al. (2021)^[^ ^203^ ^]^
	Pig	1 t of CW	Small‐scale intensive pig production	5956 kg	Liu et al. (2021)^[^ ^204^ ^]^
	Pig	1 animal in a 6020 population	Farm‐size pig production	382.66 kg	Li et al. (2021)^[^ ^205^ ^]^
	Pig	1 kg of LW	Standard pig production	1.55–1.78 kg	Chen et al. (2021)^[^ ^206^ ^]^
	Pig	1 kg of LW	Large‐scale pig production	3.39 kg	Zhou et al. (2018)^[^ ^207^ ^]^
Cuban	Pig	50 kg of LW in 1 year	Food waste	88.3785–220.4425 kg	Alba‐Reyes et al. (2023)^[^ ^208^ ^]^
	Pig	50 kg of LW or equivalent livestock unit	Genetic farms, multiplier farms, and production farms	6.87–9.65 kg	Reyes et al. (2019)^[^ ^209^ ^]^
Denmark	Pig	1 kg of CW at the slaughterhouse gate	Changes over the last 10 years	2.7–4.0 kg	Dorca‐Preda et al. (2021)^[^ ^210^ ^]^
Europe	Pig and chicken	25 kg bag in feed at 250 g per t of feed	A citrus extract feed additive	Reducing 6 t and 5 t for broilers and fattening pigs, respectively	Bui et al. (2023)^[^ ^211^ ^]^
	/	1 ton of feed ingredient	Poultry fat, poultry by‐product meal, and steam‐hydrolyzed feather meal	666, 726, and 597 kg	Campos et al. (2020)^[^ ^212^ ^]^
	Pig	1 kg of LW	Different breeds	7.19 kg	Monteiro et al. (2019)^[^ ^199^ ^]^
	Pig	A basket of two products: 1 kg of LW ‐ suckler cow calves + 1 kg of LW ‐ pigs	An integrated mixed crop‐livestock system with a green biorefinery	19.6 kg	Parajuli et al. (2018)^[^ ^213^ ^]^
Iceland	Chicken	1 kg of CW and 1 kg of eggs	Standard chicken production	2.82 kg for CW and 2.38 kg for eggs	Guojónsdóttir et al. (2025)^[^ ^214^ ^]^
Italy	Pig	1 kg of crude protein in biomass silage from the farm	Barley, rye, and sorghum	7.43, 6.33, and 4.45–7.38 kg	Noya et al. (2018)^[^ ^215^ ^]^
Japan	Broiler chicken	1 kg of LW	Low‐protein diet and litter incineration	1.86 kg	Ogino (2021)^[^ ^216^ ^]^
Mexico	Pig	1 finished pig weighing 124 kg	Farm‐size intensive pig production	538.62 kg	Giraldi‐Diaz et al. (2021)^[^ ^217^ ^]^
Netherlands	Pig	1 kg of BWG	Rapeseed meal and waste‐fed larvae meal	1.45–1.67 kg	van Zanten et al. (2018)^[^ ^218^ ^]^
Norway	Pig	1 kg of CW	Standard pig production	2.34–2.99 kg	Bonesmo and Enger (2021)^[^ ^219^ ^]^
Poland	/	1 Mg of product (rapeseed)	Winter rape production	940–980 kg	Baum and Bienkowski (2020)^[^ ^220^ ^]^
Serbia	Pig	1 kg of LW	Standard pig production	1.99–5.5 kg	Djekic et al. (2021)^[^ ^221^ ^]^
South Korea	Chicken	1 kg of CW	Standard chicken production	4.08 kg	Kang et al. (2025)^[^ ^222^ ^]^
Spain	Pig	1 kg of LW	Organic livestock farms and conventional farms	2.94–4.16 kg	Horrillo et al. (2020)^[^ ^223^ ^]^
	Pig	1 kg of LW at the farm gate	The Iberian traditional pig production	3.4 kg	Garcia‐Gudino et al. (2020)^[^ ^224^ ^]^
UK	Pig	1 kg of LW at the farm gate	Changes over the last 18 years	2.41 kg	Ottosen et al. (2021)^[^ ^225^ ^]^
	Pig	Operation of the farm over a 7‐year rotation period	Integrated organic farming	1876 t–2372 t	Jeswani et al. (2018)^[^ ^226^ ^]^
USA	Pig	1 finished pig weighing 130 kg	DDGS, food waste, and low protein and synthetic amino acids supplementation	354.1, 319.9, and 324.6 kg	Shurson et al. (2022)^[^ ^227^ ^]^
	/	1 lb diet at the feed production stage	DDGS	0.242 kg	Haque et al. (2022)^[^ ^228^ ^]^
	Pig and chicken	1 kg of LW	Soybean meal, DDGS, and synthetic amino acids supplementation	400, 869, and 2714–6789 g	Benavides et al. (2020)^[^ ^229^ ^]^
	Pig	1 kg of LW at the farm gate	Ractopamine	3.59 kg	Bandekar et al. (2019)^[^ ^230^ ^]^

^a)^ Sources are partially retrieved, screened, and summarized from Hu. (2025).^[^ ^231^ ^]^ BWG: body weight gain; CW: carcass weight; DDGS: distillers dried grains with solubles; GWP: global warming potential; LW: live weight.

**Figure 2:**
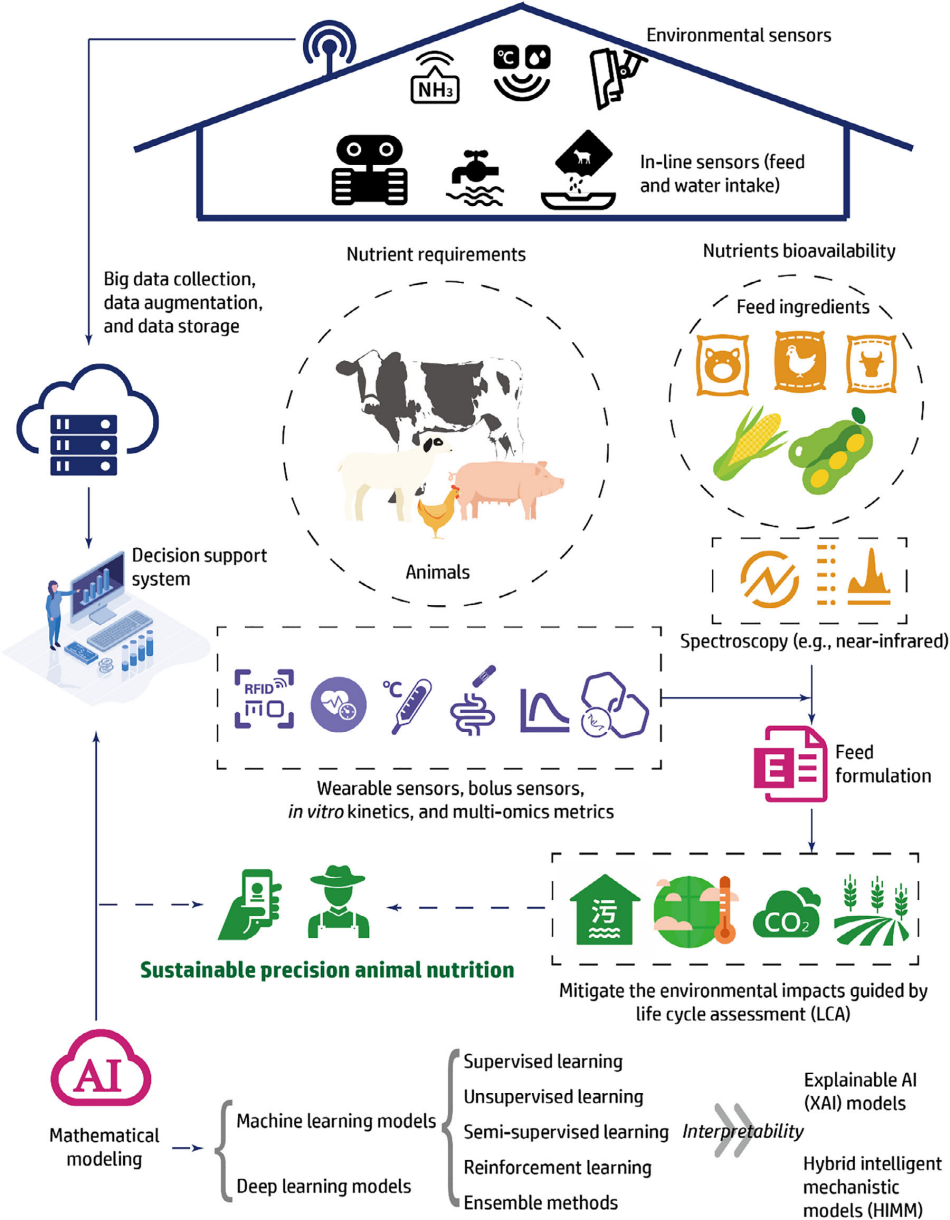
Schematic of achieving sustainable precision animal nutrition relying on intelligent data acquisition with sensors, advanced and explainable mathematical models, and life cycle assessment.

When conducting the LCA research, the database inventory data may not be directly used due to discrepancies in the specific situations of the livestock industry and feed ingredient cultivation in different countries and regions. Referring to data from other countries may affect the accuracy of the calculation results and thus lead to uncertainties in the research model. Therefore, it is necessary to establish the national or regional featured databases for environmental footprints of feed ingredients based on the actual situation of each country or region to lay the foundation for optimizing location‐specific feed formulation algorithms. In addition, optimizing and introducing new LCA application models and algorithms are also a future research direction, such as Attributional Life Cycle Assessment (ALCA) and Consequential Life Cycle Assessment (CLCA),^[^ ^105^ ^]^ which may better promote the precise assessment of environmental benefits in livestock production. The overall schematic of achieving sustainable precision animal nutrition and the related main techniques are shown in **Figure** **2**.

## 6 Case Studies of the Big Data and AI‐Powered Animal Nutrition Modeling

### 6.1 Precision Pig Nutrition and Feeding Driven by Model‐Data Fusion

**Figure 3:**
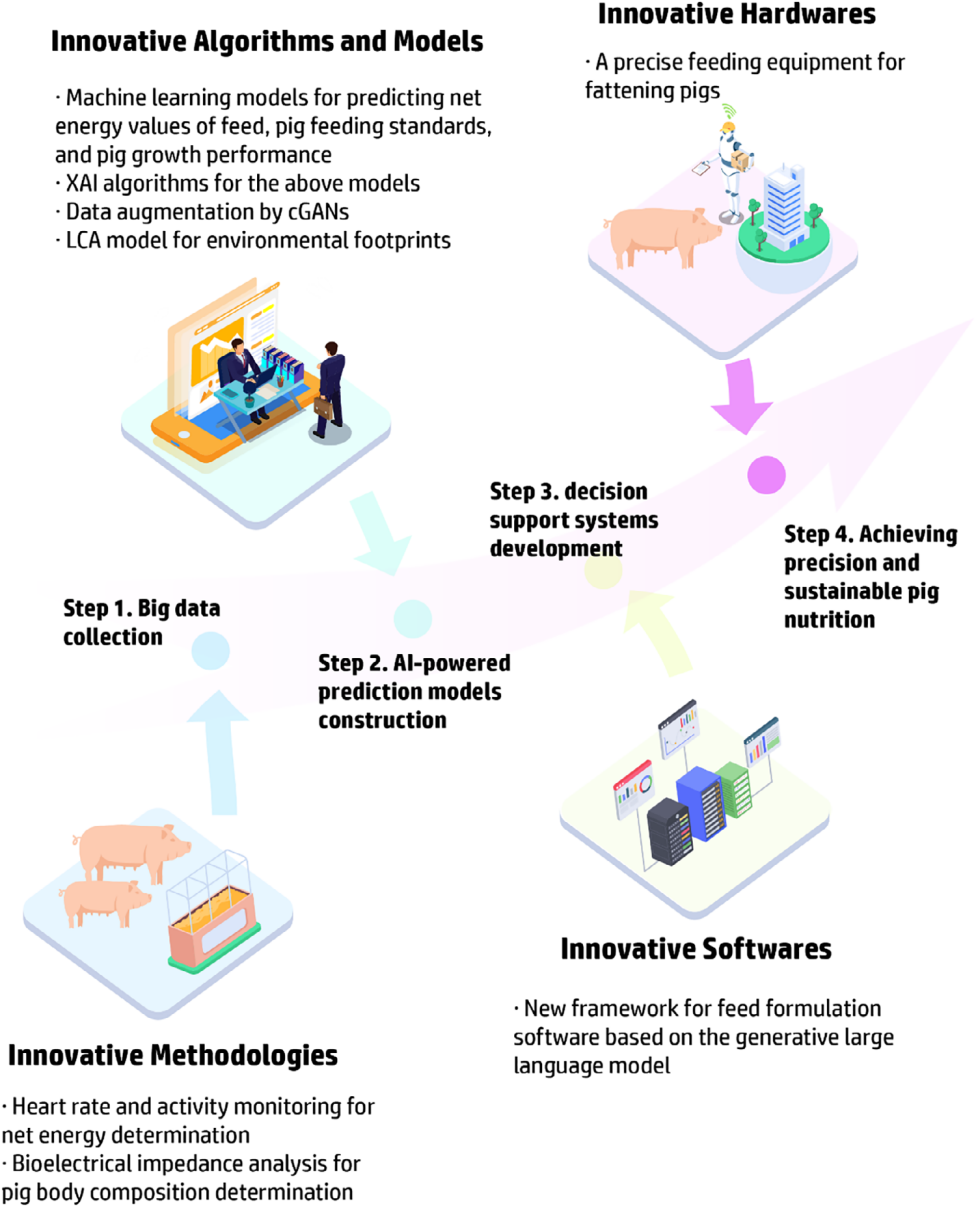
Schematic of the precision pig nutrition and feeding system driven by model‐data fusion developed by our research group and the key innovations. AI: artificial intelligence; cGANs: conditional generative adversarial networks; LCA: life cycle assessment; XAI: explainable artificial intelligence.

As the world's largest country for pig production, China has long faced challenges such as feed resource shortages, low feed utilization efficiency, and labor shortages. Given this situation, the development of precision animal husbandry, especially in pig production, has become urgent in China. Regarding the intelligent and precision nutrition of pigs, our research team (Ministry of Agriculture and Rural Affairs Feed Industry Centre, China Agricultural University) has carried out innovative explorations in methodology, algorithms, models, software, and hardware, which correspond to the following four steps: big data collection, AI‐powered prediction models construction, decision support systems (DSS) development, and finally achieving sustainable precision pig nutrition. In terms of methodology, we have proposed a low‐cost method for determining the NE value of pigs based on heart rate and activity monitoring,^[^ ^106^ ^]^ as well as a non‐destructive and rapid detection method for body compositions of pigs using bioelectrical impedance analysis (BIA). These methods reduce the cost by at least 100 times for feed nutrient value evaluation and animal nutrient requirement determination. In terms of algorithms and models, we have applied machine learning and XAI algorithms to the field of pig nutrition, establishing K‐means clustering models for predicting the NE value of feed ingredients, K‐nearest neighbor models for improving the feeding standards of pigs, as well as ANN models for predicting pig growth performance.^[^ ^6^, ^107^, ^108^ ^]^ These models have significantly improved prediction accuracy compared to the traditional simple linear regression models, and the corresponding XAI algorithms help to identify the key influential factors and their interactions. To address the issue of limited data, our team has also explored data augmentation algorithms based on generative algorithms, including conditional generative adversarial networks (cGANs). Faced with the unclear environmental impact of the pig industry in China under the “dual carbon” target, our team has adopted a LCA model to evaluate the environmental impact of different protein feed ingredients and energy evaluation systems on pork production and explored the multi‐objective optimization algorithm for pig feed formulation,^[^ ^100^ ^]^ thus providing essential data and tool support for the development of environmentally friendly pig production in China. In terms of software, based on the accumulated databases, our team has innovatively established a new framework for feed formulation software based on the generative large language model with the name “FeedMaaS”, offering new ideas for the promotion of feed formulation software in China. Regarding hardware, our team has integrated the above technologies to develop a dynamic nutrition data collection system and precise feeding equipment for fattening pigs. Compared with traditional feeding equipment, this system can reduce feed consumption by more than 12%, achieving customized feed formulations and precise feeding for pigs (unpublished data). A schematic of this case is illustrated in **Figure** **3**.

### 6.2 Precise Utilization of Alternative Feed Ingredients to Achieve Sustainable Animal Production

Conventional feed ingredients include grains and protein‐rich crops, such as corn, wheat, and soybeans, which are increasingly associated with high expenses, resource depletion, and environmental pressure. The world is facing a conflict between feeding a growing population and the scarcity of feed sources, leading to increased interest in alternative feed ingredients. These unconventional ingredients may offer environmentally sustainable ways to provide essential nutrients to livestock. Alternative feed ingredients have shown great potential as sustainable sources of protein, energy, and bioactive compounds in feed, thereby reducing competition between humans and livestock for edible crops. Moreover, alternative feed ingredients can also contribute to the circular economy by utilizing waste disposal, thus reducing the environmental footprint of animal production.^[^ ^109^ ^]^ Precision animal nutrition, facilitated by big data and AI‐powered modeling, may enable a more dynamic evaluation of the available nutrients in feed and the nutrient requirements of animals, leading to a movement from traditional phase‐feeding systems to real‐time diet adjustments based on sensor data. Therefore, the application of mathematical models enables the precise integration of alternative feed ingredients into diets, thereby directly contributing to the sustainability goals of animal husbandry. Alternative feed ingredients that have received wide attention in recent years include insect proteins (e.g., black soldier fly, yellow mealworm, housefly, grasshopper, and crickets), algae and seaweed, single‐cell proteins (SCPs, e.g., bacteria protein, fungi protein, and yeast protein), and industrial byproducts and food waste (e.g., fruit pomace).^[^ ^110^ ^]^ The application of some representative alternative feed ingredients in livestock and poultry feed is summarized in **Table** **5**, which includes the summary of their nutritional values, optimal inclusion level, and feeding effects used in poultry, pig, or ruminant diets, as well as the challenges for their industrial utilization. Future promising research directions on alternative feed ingredients may include: determining the available nutrient contents and the optimal inclusion levels or processing factors of alternative feed ingredients suitable for different animal species on site through new and rapid analytical methods (e.g., in combination with mathematical models such as transfer learning models), developing new strategies and techniques to improve the palatability and reduce the anti‐nutritional factors containing in alternative feed ingredients, adopting low‐cost preservation methods (e.g., dehydration, ensiling) to extend the shelf life and availability of seasonal alternative feed ingredients, and creating new feed ingredients with enhanced nutritional properties using technologies such as computational biology and synthetic biology.^[^ ^111^ ^]^ Overall, with the construction of more sophisticated AI‐powered models, the integration of alternative feed ingredients with other precision animal nutrition technologies is a key future direction that can enable data‐driven and sustainable livestock management.

**Table 5:** The application of some representative alternative feed ingredients in livestock and poultry feed.

Type of ingredients	Nutritional values^a)^	Optimal inclusion level in diet	Feeding effects on animals	Challenges for industrial utilization	Refs.
**Insect proteins**					
Black soldier fly (BSF)	High crude protein content (40–55%); high crude fat content (11–40%); rich in lauric acid and chitin; balanced amino acids (lysine, leucine) similar to soybean meal.	**Poultry**: up to 10–15% DM (broilers/layers); Up to 100% soybean meal replacement possible, but lower levels preferred for growth. **Pig**: up to 10% DM (growing pigs); higher levels require defatting. **Ruminant**: 30% DM as milk replacer.	**Poultry**: ↑growth performance and feed efficiency; ↑immune function (antimicrobial peptides); ↑meat quality (redness); no adverse effects on health. **Pig**: no negative impact on growth performance and carcass traits; ↓FCR; possible gut health benefits. **Ruminant**: similar kid performance to goat milk as milk replacer;↓OM digestibility due to chitin; lab isolates show probiotic potential.	High production costs; regulatory frameworks are often limited or restrictive (e.g., substrate restrictions); quality control and safety concerns (microbial contamination, allergens, purines); consumer acceptance and willingness to pay.	Astuti and Wiryawan (2022)^[^ ^232^ ^]^; Lu et al. (2022)^[^ ^233^ ^]^; Shah et al. (2022)^[^ ^234^ ^]^; van Huis et al. (2024).^[^ ^235^ ^]^
Yellow mealworm (YMW)	High crude protein content (40–60%); high crude fat content (20–40%); rich in chitin; high digestible AA profile (lysine, methionine, threonine).	**Poultry**: 5–15% DM. **Pig**: 5–10% DM.	**Poultry**:↑body weight gain and feed efficiency (↓FCR); no adverse effects on carcass traits;↑egg weight and shell thickness (layers); chitin enhances immune function. **Pig**: ↑growth performance and protein utilization; ↑ileal digestibility of essential Aas; ↓diarrhea incidence (weaners); defatting advised for high‐fat diets. **Ruminant**: potential methane reduction (chitin effect); possible use in young ruminants.	The same as above.	Belhadj Slimen et al. (2023)^[^ ^236^ ^]^; Fu et al. (2025)^[^ ^237^ ^]^; Hong et al. (2020)^[^ ^238^ ^]^; Shah et al. (2022)^[^ ^234^ ^]^; Syahrulawal et al. (2023)^[^ ^239^ ^]^; van Huis et al. (2024).^[^ ^235^ ^]^
Housefly (HFL)	High crude protein content (≈60%).	**Poultry**: up to 8% in broiler diets, replacing soybean meal without adverse effects.	**Poultry**: no significant differences in growth performance, carcass traits, or meat quality compared to soybean meal; slight ↓ in breast muscle shear force. **Ruminant**: ↑digestibility of crude protein; ↓methane emissions.	The same as above.	Belhadj Slimen et al. (2023)^[^ ^236^ ^]^; Elahi et al. (2020)^[^ ^240^ ^]^; Fu et al. (2025).^[^ ^237^ ^]^
Grasshopper	High crude protein content (≈50%).	**Poultry**: up to 100% in poultry diets, replacing fishmeal (usually 5–10% inclusion).	**Poultry**: ↑growth performance, feed efficiency, and nutrient retention.	The same as above.	Belhadj Slimen et al. (2023)^[^ ^236^ ^]^; Shah et al. (2022)^[^ ^234^ ^]^; van Huis et al. (2024).^[^ ^235^ ^]^
Crickets	High crude protein content (≈65%).	**Poultry**: up to 25% in quail/layer diets, replacing soybean meal.	**Poultry**: ↑egg production, feed conversion ratio, and immune response (e.g., increased IgG levels) in layers; no negative impact on meat quality. **Ruminant**: ↓methane production during production.	The same as above.	Belhadj Slimen et al. (2023)^[^ ^236^ ^]^; Shah et al. (2022)^[^ ^234^ ^]^; van Huis et al. (2024)^[^ ^235^ ^]^; Fu et al. (2025).^[^ ^237^ ^]^
**Algae and seaweed**	High protein content (20–70%); rich in omega‐3 polyunsaturated fatty acids (EPA/DHA), minerals (I, Ca, Fe), antioxidants (carotenoids, polyphenols), and vitamins (A, E, B_12_).	**Poultry**/**Pig**: 1—3% DM. **Ruminant**: up to 5% DM.	**Poultry**: ↑meat oxidative stability, reduced pathogens (*Salmonella*). **Pig**: ↑gut health, ↓lipid oxidation. **Ruminant**: ↑milk omega‐3, ↓enteric methane (up to 80%).	High production costs (specialized equipment, energy‐intensive processes); limited large‐scale production infrastructure; variability in nutritional composition; storage and shelf‐life limitations.	Corino et al. (2019)^[^ ^241^ ^]^; Morais et al. (2020).^[^ ^242^ ^]^
**Single‐cell proteins (SCPs)**	High protein content (40–80%); rich in essential amino acids (lysine, methionine), vitamins (B complex), minerals; low fat content; nucleic acids (4–16%) require processing.	**Poultry**/**Pig**: 5–15% DM. **Ruminant**: up to 10% DM.	**Poultry**: ↑growth rate and immune response; ↓FCR. **Pig**: ↑nutrient digestibility; balanced gut microbiota. **Ruminant**: ↑microbial protein synthesis; ↓ammonia emissions.	High production costs (capital investment, energy‐intensive processes); process scalability challenges (dilute solutions, expensive concentration methods, water removal); quality control and safety concerns (high nucleic acid content, potential microbial toxins).	Bratosin et al. (2021).^[^ ^243^ ^]^
**Fruit pomace /food waste**	Variable protein content (5–25%); high fiber content (20–60%); rich in polyphenols and antioxidants; solid state fermentation can: ↑crude protein (up to 49%), ↓fiber, ↑antioxidants.	**Poultry**/**Pig**: 10–20% DM. **Ruminant**: 20–30% DM (with solid state fermentation).	**Poultry**: ↑feed efficiency, egg quality, and antioxidant status. **Pig**: ↑growth performance;↓cholesterol. **Ruminant**: ↑feed efficiency;↓methane (28%); ↑milk yield; solid state fermentation ↓ anti‐nutritional factors.	Nutritional variability (inconsistent profile); feed safety concerns (microbial pathogens, mycotoxins, heavy metals, plastics); logistics of collection and distribution (urban locations, cost‐prohibitive transport for high‐moisture); economic viability (seasonal availability, frequent formulation changes, specialized storage costs).	Ikusika et al. (2024)^[^ ^244^ ^]^; Kumar et al. (2024).^[^ ^245^ ^]^

^a)^ ADG: average daily gain; DHA: docosahexaenoic acid; DM: dry matter; AA: amino acid; EPA: eicosapentaenoic acid; FCR: feed conversion ratio; OM: organic matter.

## 7 Next‐Generation Models to Achieve Sustainable Precision Animal Nutrition

In our perspectives, the development of mathematical models for animal nutrition in the future will trend toward becoming more accurate and intelligent, with large language models and embodied AI robots as future directions.

### 7.1 Decision Support Tools Relying on Large Language Models and Multi‐Agents for Knowledge Augmentation

**Figure 4:**
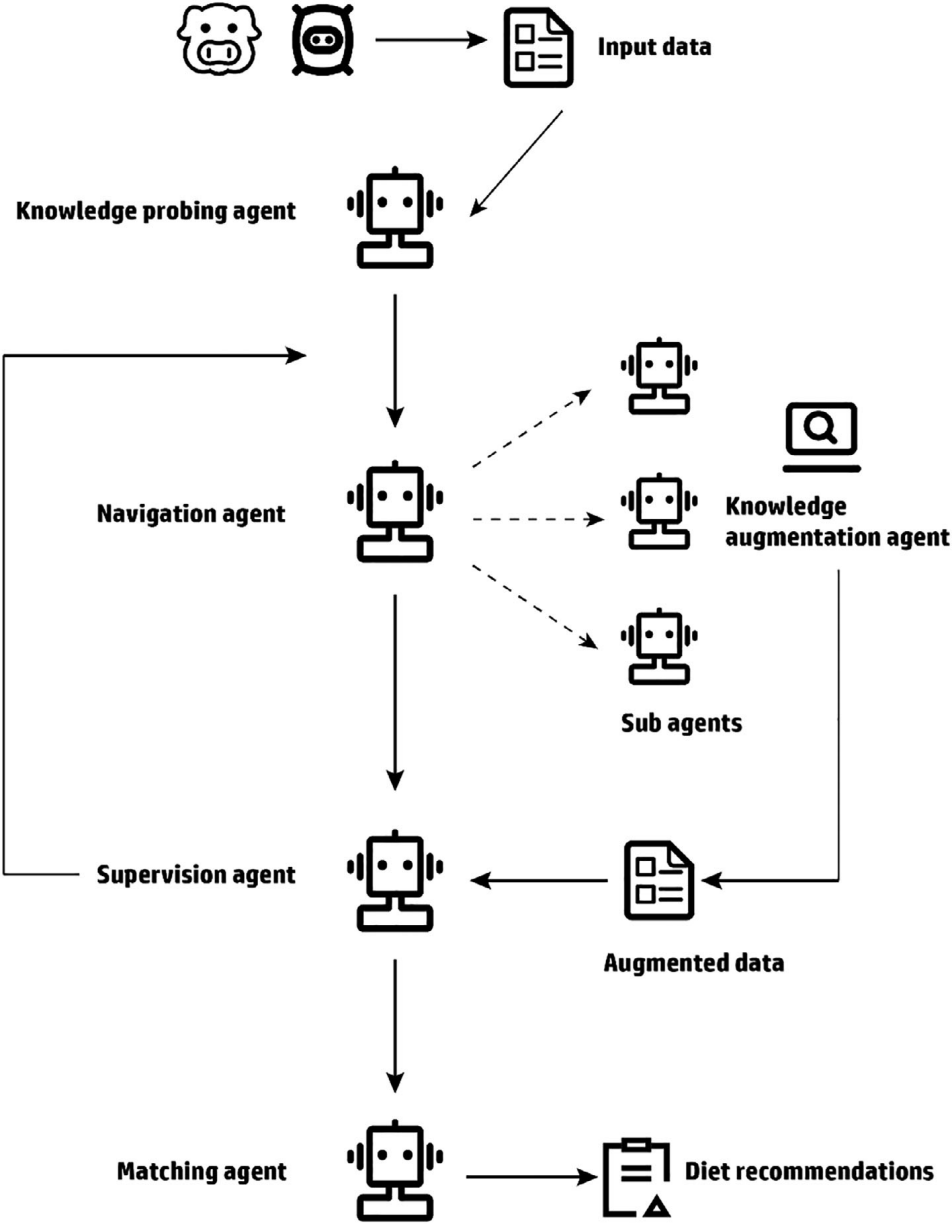
A framework of the multi‐agents for knowledge augmentation adapted to precision animal nutrition (a revised version based on the original figure by Shi et al.^[^ ^113^ ^]^).

Decision support systems have been used in animal nutrition for a long time to assist in making management decisions. These systems have evolved from early rule‐based systems to more sophisticated tools leveraging AI and big data.^[^ ^112^ ^]^ Next‐generation DSS may be poised to incorporate advancements in large language models (LLMs) and multi‐agent AI systems to further enhance knowledge augmentation and decision‐making in sustainable precision animal nutrition. LLMs have demonstrated strong capability in natural language processing and knowledge reasoning, thus enabling them to integrate precision animal nutrition‐related knowledge and data to generate interdisciplinary knowledge graphs and provide an expert‐level basis for predicting animal nutrient requirements and optimizing feed formulations.^[^ ^58^ ^]^ However, the information learned by LLMs can be outdated or misaligned with the unique needs of certain practices. To address this limitation, a novel architecture named multi‐agents for knowledge augmentation (MAKA) has been designed in clinical medicine to enhance the patient‐matching process by dynamically integrating domain‐specific knowledge aligned with the unique context of clinical trials.^[^ ^113^ ^]^ Similarly, this framework can be adapted to precision animal nutrition to better optimize dietary recommendations by addressing domain‐specific knowledge gaps with the following four main components: the knowledge probing agent can identify deficiencies in the input data to decide whether data augmentation is required; the navigation agent acts as a coordinator to guide sub‐agents based on identified gaps (e.g., feed ingredients database sub‐agent, animal nutrient requirement modeling sub‐agent, and feed formulation sub‐agent; if needed, the knowledge augmentation agent can be involved to enrich data by integrating different sources); the supervision agent can validate the augmented outputs against predefined rules; and the matching agent is the final decision‐maker to propose the recommended diets. A framework of this system is illustrated in **Figure** **4**. This system can continuously iterate model parameters through reinforcement learning mechanisms and protect farm data privacy using federated learning. The decentralized structure of the system may adapt to complex scenarios of farms with different sizes, significantly improving precision animal nutrition and production efficiency and reducing environmental burdens, and has the potential to ultimately promote the sustainable and intelligent transformation of livestock production.

### 7.2 Intelligent Feeding Enabled by Embodied AI Robots

Embodied AI robots, which deeply integrate physical manipulation capabilities with environmental intelligence, may reshape the technological paradigm of precision feeding. Unlike data‐driven machine learning methods, embodied learning focuses on robot learning through physical interaction with the environment and perceptual feedback, making it especially suitable for robotic manipulation.^[^ ^114^ ^]^ When integrated with LLMs, the large‐language‐model‐enabled robot framework, such as GPT‐4 and retrieval‐augmented generation infrastructure, can enable robots to complete long‐horizon tasks in unpredictable settings.^[^ ^115^ ^]^ The development of embodied AI robots for precision animal nutrition may include various types as follows: the feeding robots can dynamically adjust feeding amounts and timing according to real‐time changes in animal performance and behavior patterns with optimized strategies and algorithms based on deep reinforcement learning; the veterinarian robots can provide early warnings of nutritional and metabolic diseases through non‐contact body measurement and gait analysis based on computer vision; and the multi‐robot collaborative operation networks can achieve the distributed precision management in large‐scale farms. Moreover, through real‐time data interaction with digital twin platforms, the robot system can establish a closed‐loop control from perception, decision, and execution to feedback. Additionally, their autonomous navigation and cleaning functions can reduce biosecurity risks associated with human–animal contact. Combined with blockchain technology, the embodied robots may achieve traceable precision nutrition management throughout the production process, providing technical support for improving animal welfare and ensuring food safety, and potentially making livestock production enter a real intelligent era.

## 8 From Farm to Clinic: Precision Animal Nutrition as a Blueprint for Personalized Human Health Interventions

The technological accumulation of animal nutrition models provides important paradigm references for human precision nutrition research, including making personalized nutrition plans and acting as a reference for human health interventions.

### 8.1 Making Personalized Nutrition Plans Using Knowledge from Tailored Individual Animal Diet

Achieving precision feed formulation by dynamically monitoring individuals’ physiological characteristics (such as body weight, growth stage, and health status) and combining big data analysis in precision animal nutrition offers cross‐boundary migration innovation for personalized human nutrition plans. Moreover, the “precision feeding” model based on metabolomics and gut microbiota detection and AI‐powered models in animal nutrition has gradually matured.^[^ ^116^, ^117^ ^]^ This dynamic adjustment and multidimensional evaluation mechanism can be transferred to the field of precision human nutrition. For example, regarding technological tool collaboration, the IoT sensors used in animal nutrition (such as real‐time monitoring of feed intake and growth performance) are technologically similar to wearable devices for humans (dynamic blood glucose monitoring analysis).^[^ ^118^ ^]^ Regarding the innovation of algorithms and modeling techniques, the “genetic characteristics‐nutrient requirements‐production performance” correlation model established in animal research may provide methodological inspiration for precision human nutrition. Cross‐boundary migration in nutrition science reveals the universal laws of biological nutritional response mechanisms, but two core issues must be addressed. The first issue is that the complexity of human social behavior factors (such as dietary culture and psychological preferences) far exceeds that of animal models. The second issue is that a cross‐species data standardization system has not yet been established. In addition, ethical issues related to this cross‐boundary migration must also be considered in advance.

### 8.2 Exploring Fundamentals of Nutrigenomics and Human Health Interventions Using Precision Animal Models

Nutrigenomics is the area that uses molecular tools to search, access, and understand the responses of a diet supplied to individuals or population groups to elucidate how the bioactive components in the diet may affect gene expression.^[^ ^119^, ^120^ ^]^ The involvement of nutritional factors in activating and regulating key genes is associated with diseases ranging from inflammation to cancer.^[^ ^119^ ^]^ It is not easy to conduct nutrigenomics studies directly on the human body. However, the transfer learning technology provides alternatives by transferring knowledge from animal models to humans. For example, the piglet model provides an ideal human infant nutrition research platform.^[^ ^121^ ^]^ Therefore, animal nutrigenomics research can provide important technological collaboration pathways for human precision nutrition by analyzing the interaction mechanisms between genetic variation and nutritional metabolism. For instance, the research paradigm of “gene‐nutrition” interaction in animals has direct reference significance for preventing and controlling chronic diseases in humans: human nutrigenomics can quickly locate key gene targets affecting nutrient metabolism through cross‐species conserved metabolic pathway analysis. In addition, the mature application of gene editing technologies such as CRISPR in animal models can also open up new dimensions for human nutrition research. Regarding industrialization transformation mechanisms, the full‐chain research and development system of “gene editing‐functional diets‐health monitoring” in animal husbandry also has reference value for human medicine.

The current challenges in this field are establishing cross‐species nutrigenomics databases and developing causal reasoning models based on knowledge graphs to transform key regulatory networks in animal experiments into human nutrition intervention targets.^[^ ^122^ ^]^ This technological collaboration can shorten the transformation cycle from basic research to clinical application and reveal the evolutionary laws of nutritional adaptation through comparative genomics, laying the foundation for developing universal nutrition strategies based on evolutionary medicine. In the future, establishing a trinity research system of “animal models‐organoids‐human trials” is expected to achieve precise prediction of human nutrient requirements and the prevention of metabolic diseases from the source.

## 9 Limitations and Prospects of the AI‐Powered Modeling

The AI‐powered models will bring a significant paradigm shift in animal science research, facilitating precise and sustainable feed practices. However, like other fields, limitations and challenges still exist for deploying AI‐powered models in practice, including issues related to ethics, data privacy, cognition, and hardware and infrastructure requirements.^[^ ^123^, ^124^ ^]^ AI‐powered models rely heavily on advanced algorithms; as a result, they are susceptible to algorithmic bias, including biases in training data, model design, and those introduced by human developers. Algorithmic bias represents a kind of societal injustice, making discrimination more pervasive and difficult to challenge. An AI‐powered model also relies heavily on big data to realize its full potential, and the quality, diversity, and relevance of the data fundamentally determine the accuracy and generalizability of AI model training. The data collection process may also frequently involve sensitive personal information.^[^ ^125^ ^]^ Without sufficient safeguards, the data used for AI model development is easily misused. Moreover, the rapid evolution of AI technology outpaces regulatory and governance frameworks, and the lack of clear guidelines may lead to compliance issues.^[^ ^126^ ^]^ Although new laws have been created in the European Union, the United States, and China, the governance of AI technologies still faces significant uncertainties in law and ethics.^[^ ^123^ ^]^ Therefore, more flexible and adaptive regulatory mechanisms that can evolve with the AI technology should be developed. Furthermore, in an era with the popularity of Large Language Models (LLMs), it seems more straightforward for models to identify and imitate linguistic patterns. However, those advanced models do not truly “understand” the input of users, and are still struggling to apply knowledge beyond their training, often leading to “hallucinations”. There is still a long way to go to train a model with human‐like logic and genuine creative capacity. Lastly, training large AI models demands a large amount of computing power and energy, which also means high costs and a significant carbon footprint. Additionally, distributed AI workloads require high‐bandwidth and low‐latency interconnects, as single points of failure or signal integrity issues can cause model training to stall.^[^ ^127^ ^]^ The high requirements in hardware and infrastructure make it more expensive and difficult for advanced AI models development, especially for startups and smaller teams, which is a significant limitation to inspiring innovation in the AI area. Although the above limitations of AI‐powered models may persist for some time, we should remain confident in the progress of AI technology, which will ultimately lead to excellent solutions for these challenges.

## 10 Conclusion

Animal nutrition models driven by big data and artificial intelligence are triggering industrial changes: traditional, simple linear models are transforming toward precision and intelligence, providing solutions against global challenges such as feed shortages and climate change. The future development directions of animal precision nutrition may include lower cost control, more precise individual prediction, and deeper integration with artificial intelligence technologies. In addition, future research should focus on upgrading large language models and multi‐agent systems and developing precision‐feeding embodied robots. It should also pay more attention to collaborative research between animal nutrition and human precision nutrition, supporting human health and a better life with efficient, intelligent, and sustainable livestock production.

## Conflict of Interest

The authors declare no conflict of interest.
